# Characterizing the Contribution of Indoor Residential Phthalate and Phthalate Alternative Dust Concentrations to Internal Dose in the US General Population: An Updated Systematic Review and Meta-Analysis

**DOI:** 10.3390/ijerph20166589

**Published:** 2023-08-17

**Authors:** Sashoy G. Milton, Rachel A. Tejiram, Rashmi Joglekar, Kate Hoffman

**Affiliations:** 1 Nicholas School of the Environment, Duke University, Durham, NC 27710, USA; sashoy.milton@duke.edu; 2 Nash Family Department of Neuroscience, Icahn School of Medicine at Mount Sinai, New York, NY 10029, USA; rachel.tejiram@icahn.mssm.edu; 3 Earthjustice, Toxic Exposure and Health Program, Washington, DC 20001, USA

**Keywords:** phthalate, phthalate alternative, meta-analysis, reverse dosimetry, indoor residential dust, systematic review

## Abstract

Diet is the primary exposure pathway for phthalates, but relative contributions of other exposure sources are not well characterized. This study quantifies the relative contribution of indoor residential dust phthalate and phthalate alternative concentrations to total internal dose estimated from the National Health and Nutrition Examination Survey (NHANES) urinary metabolite concentrations. Specifically, median phthalate and phthalate alternative concentrations measured in residential dust were determined by updating a pre-existing systematic review and meta-analysis published in 2015 and the attributable internal dose was estimated using intake and reverse dosimetry models. Employing a predetermined search strategy, 12 studies published between January 2000 and April 2022 from Web of Science and PubMed measuring phthalates and phthalate alternatives in residential dust were identified. From the data extracted, it was estimated that dust contributed more significantly to the internal dose of low-molecular weight chemicals such as DEP and BBP when compared to high-molecular weight chemicals such as DEHTP. Additionally, findings showed that the chemical profile of residential dust is changing temporally with more phthalate alternatives being detected in the indoor environment. Future studies should seek to characterize the contribution of dust to an overall phthalate and phthalate alternative intake for individuals who have higher than normal exposures.

## 1. Introduction

*Ortho-*phthalate esters and alternatives to *ortho-*phthalate esters (referred to as phthalates and phthalate alternatives hereafter) are commonly added to plastics to increase their flexibility, transparency, durability, and longevity [1]. They are also commonly used as solvents, stabilizers, and additives in cosmetics, medical devices, household, and personal care products [2,3]. 

Phthalates are a high-production chemical class with more than 470 million pounds produced or imported into the United States each year [4]. Over the last few decades, exposure to phthalates has been linked to serious health effects, including reproductive toxicity and neurodevelopmental harm, which has led to a ban of the use of several phthalates in children’s products by the U.S. Consumer Product Safety Commission [5,6,7,8,9]. The concern surrounding the safety of the use of phthalates in consumer products had led to an increase in the use of phthalate alternatives such as citrates, terephthalates and adipates in consumer products, but less is known about their toxicity and distribution in the indoor residential environment [10,11]. However, due to their extensive use in a variety of consumer products for decades, phthalates are still found in relatively high concentrations in the indoor environment [12]. Since phthalates bind weakly to the materials they are added to, they easily leach, migrate, or off-gas into environmental media. Studies show that diet is likely the main source of human exposure to phthalates [13];, however, other sources of exposure such as indoor air and dust can contribute significantly to an individual’s daily phthalate intake depending on their age and lifestyle [14,15,16].

Starting in December 2019, EPA launched risk evaluations for seven phthalates—dibutyl phthalate (DBP), benzyl butyl phthalate (BBP), bis(2-ethylhexyl) phthalate (DEHP), diisobutyl phthalate (DiBP), dicyclohexyl phthalate (DCP), diisodecyl phthalate (DIDP), and diisononyl phthalate (DiNP) under the Toxic Substances Control Act (TSCA). When evaluating the human health risk posed by these phthalates, TSCA requires EPA to examine all reasonably foreseeable routes and pathways through which humans are exposed, which must include exposures occurring via residential dust [17]. However, few studies provide information on the contributions of individual exposure sources and routes to a person’s total internal phthalate exposure. Further, given the increase in the use of phthalate alternatives, it is also vital to characterize the distribution of phthalate alternatives in the environment and their contribution to an individual’s exposome. 

On average, people living in the US spend 90% of their time indoors and nearly 70% in residential environments [18]. The amount of time spent indoors is even higher for individuals who are highly susceptible to the impacts of pollution, such as young children and people with underlying health conditions, highlighting the importance of the home environment when characterizing phthalate and phthalate alternative exposures. In the indoor environment, phthalates are known to partition between dust, air, and exposed surfaces such as skin [19], leading to potential human exposure through inhalation, ingestion, and dermal absorption [20,21,22]. Studies have shown that the analysis of household dust provides a snapshot of the types of phthalates and phthalate alternatives present in the indoor environment [23,24]. As such, measured or modeled dust concentrations can be used to estimate the partition of chemical substances between the air, airborne particles, and other various surfaces, thereby allowing for the characterization of total residential exposures and related intake [25,26,27,28]. Furthermore, infants and young children are more exposed to chemicals found in dust when compared to other age groups, since they crawl and play on the floor and are more likely to engage in hand-to-mouth behavior [12,21,29]. As such, understanding phthalate and phthalate alternative levels present in dust can provide critical insights into an individual’s phthalate and phthalate alternative exposures during early life. 

This systematic review and meta-analysis synthesize evidence to quantify levels of phthalates and phthalate alternatives in indoor residential settled dust levels across the US general population. Building on evidence presented by Mitro et al. in 2015 [5], we methodically screened relevant studies to obtain a central point estimate (median) of residential dust phthalate and phthalate alternative concentrations. The point estimates of the chemical substances included in the meta-analysis were then used to obtain daily intake rate estimates for the relevant exposure routes for toddlers (3 to <6 years) and adults (≥21 years). Finally, we used a reverse dosimetry model to infer daily intake estimates from the total measured urinary metabolites for each included chemical (using phthalate and phthalate alternative urinary metabolite levels reported in the National Health and Nutrition Examination Survey (NHANES)). This represented the overall phthalate and phthalate alternative internal dose in the general population irrespective of source, route, or pathway of exposure. We then compared the daily dust intake rate estimates (systematic review/meta-analysis) to the overall daily intake rate estimates (NHANES) to determine the proportion of internal phthalate and phthalate alternative dose attributable to measured indoor residential phthalate and phthalate alternative dust concentrations for the included study populations. Finally, we provided a summarized toxicity profile for each detected chemical substance to provide context for potential human health effects. These methodologies were employed to quantify the contribution of indoor phthalate and phthalate alternative dust concentration levels to internal phthalate and phthalate alternative exposure levels in the US general population.

## 2. Methods

### 2.1. Study Inclusion and Exclusion Criteria

To obtain an estimate of the median phthalate and phthalate alternative dust concentration levels in the residential indoor environment, we updated a systematic review and meta-analysis carried out and published by Mitro et al. in 2015 [5]. The preceding review carried out an exhaustive literature search of Web of Science and PubMed for dust analysis studies published from January 2000 to February 2015. Building on the previous review, we conducted a search for residential indoor dust analysis studies published from March 2015 to April 2022 using the same databases.

The studies included in the present review met the eligibility criteria if the study was peer-reviewed, dust samples were collected in the US during or after the year 1999, samples were from an indoor residential environment, collected using a vacuum cleaner (either vacuumed dust collected by study team or from an existing bag), and measured phthalates and/or phthalate alternatives. Studies were excluded if they did not include primary data, the location where study was conducted was unclear, or were not in English. 

### 2.2. Systematic Literature Search

PubMed and Web of Science were searched for all peer-viewed document types using the following search criteria, [phthalate*] AND [dust*]. The literature search was filtered by language: English and species: human. The literature identified in the database searches was imported into the web-based systematic review management software, Covidence (Veritas Health Innovation, Melbourne, Australia [30]).

### 2.3. Screening of Studies

In Covidence, the title and abstract of the imported studies were de-duplicated and then manually screened by two independent reviewers for relevancy to the pre-determined eligibility criteria. Screening consensus between reviewers was greater than 90%. Conflicts in inclusion decisions made by the reviewers were tracked in the software and were resolved through discussions with a third reviewer. Studies determined to be relevant at the title and abstract review stage by the screeners were moved to the full text review stage in Covidence. Here, the full text for the studies that were included at the title and abstract level were uploaded and independently reviewed against the eligibility criteria. Conflicts at this stage were also resolved through discussions with a third reviewer. From the previously published review, five studies met our eligibility criteria and were included at the full text review stage. To reduce the chances of missing a relevant study, we also conducted a manual review of the reference list of studies screened at the full text review stage. No additional studies were identified through this manual review. Studies that were included after full text screening advanced to the data extraction step to be included in the meta-analysis. 

### 2.4. Strength of Evidence Assessment

During data extraction, descriptive information such as duration and location of study, quality control and assurance methods used, and the presence or absence of a demographic description of the occupants of the residential environment were noted. Quantitative information, such as phthalate and phthalate alternative dust concentrations, size of dust sampling sieve, and the treatment of below-method of detection limit (MDL) measurements were also extracted. The summarized extracted information was used to support the strength of evidence assessment (see Supplementary Information (SI): Table S6).

An amended version of the strength of evidence tool for epidemiology studies described in DeLuca et al. (2021) was used to assess the strength of evidence for all the studies included in the meta-analysis [31]. The strength of evidence across the studies was independently judged by two reviewers for each of the following criteria: exposure measurement, participant selection and analysis. The reviewers assigned a rating of “good”, “adequate”, or “deficient” for each domain and recorded their reasoning for the rating assigned to each study. The results of this assessment can be found in SI: Table S10. A study was thought to have a low risk of bias if its overall risk of bias rating was either “good” or “adequate” and was thought to have high risk of bias if its overall risk of bias rating was “deficient”. Discrepancies between the judgments and ratings were resolved through discussion by the two reviewers. The impact of studies categorized as having a high risk of bias on the overall pooled phthalate and phthalate alternative concentrations was explored using a sensitivity analysis.

### 2.5. Meta-Analysis of Phthalate and Phthalate Alternative Dust Concentrations

The dust concentrations for phthalates and phthalate alternatives detected in ≥50% of samples in at least two studies were extracted for use in the meta-analysis. If samples were reported for different exposure scenarios (e.g., different seasons or in different types of homes), we reported dust measurements separately and included the measurements in the meta-analysis as a separate study record [22,32]. Therefore, we included 15 datasets as our unit of analysis in the meta-analysis.

As all the studies reported medians instead of means as the measure of center, a weighted median of medians approach was taken to estimate the pooled median phthalate and phthalate alternative concentrations [33]. Under this approach, the weights, or the frequency with which the study-specific medians appeared, were directly proportional to the number of subjects and normalized to a sum of 1. It was assumed that the weights were independent of the observed study medians and that the observed study medians were drawn from a distribution with a median equal to the population median. The weighted median of medians’ approach was implemented using the meta-median R package available on CRAN (https://CRAN.R-project.org/package=metamedian, version 0.1.6, accessed on 31 October 2022).

### 2.6. Sensitivity Analysis Exploring the Impact of Studies with a High Risk of Bias

The potential influence of studies with a high risk of bias on the estimated pooled weighted median dust concentrations for each chemical substance was explored by comparing the relative percentage point difference (PPD) between the pooled weighted median concentrations, when all studies were included and when only the studies deemed as having a low risk of bias were included in the analysis. A PPD of less than 10% was considered acceptable.

### 2.7. Daily Intake Rate Assessment from Dust Concentrations

The residential daily intake rate (ug/kg-day) for each chemical included in the meta-analysis was predicted for children (3 to <6 years) and adults (≥21 years) using the estimated and pooled median concentrations. As infants and young children are more exposed and vulnerable to chemical exposures from dust, we were interested in the difference between their daily intake rate and that of adults. To estimate the total internal exposure of phthalate or phthalate alternative levels, we relied on urinary metabolite data from the NHANES 2017–2018 survey cycle. As the 2017–2018 survey cycle did not include urine metabolite levels for children under the age of 3, we limited the age range in our study of infants/children to 3–5 years. We estimated the daily residential intake rate from indoor dust for adults and children for three exposure routes: dust ingestion, dermal absorption from the gas phase, and inhalation of air (see Table 1). Dermal absorption from dust adhered to skin was excluded, as the contribution of exposure from this route has been shown to be negligible [5,19].

A previously validated partitioning model [26] was used to predict the total air concentration for all phthalates and phthalate alternatives measured in dust, except dimethyl phthalate (DMP) and diethyl phthalate (DEP). The model assumes that equilibrium partitioning is governed by the physical–chemical process of absorption in the organic fraction of dust, making octanol a suitable chemical model for the organic matter in dust for sorption [25,26]. Simplified mass transfer models sometimes fail to account for the full range of complexities associated with the dynamic transfer of phthalate particles in the indoor environment, which can impact the prediction of air-phase phthalate concentrations from the dust phase [35]. However, the present model has been shown to correlate well (R^2^ = 0.8) between measured and predicted concentrations [26]. 

As DEP and DMP exist mostly in the gas phase, each chemical’s gas phase concentration was estimated by assuming a linear relationship between the mass fraction in settled dust and the gas phase concentration [19] (See Supplemental Information). The equations and the exposure factors used to determine individual daily intake rates for the other phthalate and phthalate alternatives from the co-occurring exposure routes can be found in Table 1.

Physiochemical properties for each chemical, including the octanol–water coefficient (K_ow_), octanol–air partitioning coefficient (K_oa_), air-water distribution ratio (K_aw_), Henry’s law constant and molecular weight, which were used to compute each intake prediction, were pulled from EPA’s EPI Suite (v4.11), as reported in Mitro et al., Schossler et al., and the EPA CompTox Dashboard [5,36,37,38,39]. In the instances where the K_aw_ for a specific chemical substance was not reported, it was calculated using the formula reported in the Supplemental Information. 

### 2.8. Total Daily Intake-Rate Estimated from Metabolite Concentrations in Urine

“Total daily dose or total daily intake-rate” was determined using phthalate and phthalate alternative urinary metabolite concentrations measured during the NHANES 2017–2018 cycle [40]. We estimated the total daily dose for each parent chemical measured in dust from their urinary metabolite concentrations using the dose reconstruction formula shown below. The NHANES biomonitoring data incorporated into the formula represent the total phthalate and phthalate alternative dose in the general population from non-specific sources for adults (21 years and older) and children (3–5 years). The dose reconstruction formula below has previously been applied in the literature [41,42,43,44,45,46]. 

Dose reconstruction formula:

$$Daily\;intake\;rate\;\left(\mathrm{ug}/\mathrm{kg}-\mathrm{day}\right)=\frac{UER\;\left(\frac{\mathrm{ug}}{\mathrm{day}*\mathrm{kg}\;BW\;}\right)*MW_{p}}{F_{UE\;}*MW_{m}},$$

where *UER* represents the body-weight-adjusted urinary excretion rate for each age group, *F_UE_* is the molar fraction that describes the sum molar ratio between the excreted amounts of the metabolites of each phthalate and phthalate alternative corresponding to the intake of the parent phthalate, *MW_p_* is the molar weight of the phthalate ester parent compound, and *MW_m_* is the molar weight of the corresponding phthalate monoester. When a phthalate or phthalate alternative had multiple measured metabolites, the sum of the intake rates across the metabolites was taken. If a metabolite shared a parent phthalate, the urinary concentration was divided in half amongst the parent molecules and then the intake rate was calculated.

The urinary excretion rate (*UER*) for each phthalate and phthalate alternative was calculated by:

$$UER\;\left(\frac{\mathrm{ug}}{\mathrm{kg}\;\mathrm{per}\;\mathrm{day}}\right)=\frac{{UE}_{crea}\;\left(\frac{\mathrm{ug}}{g_{crea}}\right)*\mathrm{CE}\;\left(\frac{g}{\mathrm{day}}\right)}{\mathrm{BW}\;\left(\mathrm{kg}\right)},$$

where *CE* is the mean creatinine excretion rate specific to the weight, height, and sex of the individuals within each age group and *UE_crea_* is the urinary creatinine-adjusted urinary phthalate or phthalate alternative concentration level at the 50th percentile. Both variables were estimated using the NHANES 2017–2018 data after accounting for the sampling design using the package “survey” [47] in the R statistical programming environment (https://CRAN.R-project.org/web/packages/survey, version 4.1-1, assessed on 9 February 2023). *CE* for each age group was calculated using a set of equations proposed by Mage et al. [48,49] using the following variables from the NHANES dataset: RIAGENDR, BMXHT, RIAAGEYR, and BMXWT. *UE_crea_* was determined using the following formula from a study of Danish children 6–11 years of age, carried out by the Ministry of Environment and Food of Denmark Environmental Protection Agency (EPA) [50]:

$$UE_{crea}\;\left(\frac{\mathrm{ug}}{g_{crea}}\right)=\frac{UC\;\left(\frac{\mathrm{ug}}{L}\right)*1000\;\left(\frac{\mathrm{mg}}{g}\right)}{UC_{crea\;}\left(\frac{\mathrm{mmol}}{L}\right)*\;MW_{crea}\left(\frac{\mathrm{mg}}{\mathrm{mmol}}\right)},$$

where *UE* is the urinary excretion of the urinary phthalate and phthalate alternative metabolites per gram of creatinine for each age sub-population, *UC* is the median-measured urinary concentration of each compound, *UC_crea_* is the median age-group-specific urinary concentration of creatinine, and *MW_crea_* is the molecular mass (114 mg/mmol) for creatinine. The median age-group-specific urinary concentration of creatinine was obtained from the adjusted NHANES 2017–2018 survey data. A creatinine-based method was used to predict the glomerular filtration rate, as it included various physiological parameters for the age groups of concern and had the relevant parameters available in the NHANES dataset. 

In the dose reconstruction formula, the molar fraction, (*F_UE_*), which describes the molar ratio between the excreted amounts of the metabolites of each phthalate and phthalate alternative, was determined based on the proportional relationships previously assumed in the literature [46,51,52,53,54,55,56]. A 1:1 stoichiometry of the parent to metabolite molecule was assumed unless otherwise indicated by simplified molecular-input line-entry system (SMILES) descriptors available for the parent and metabolites. Our approach assumes a steady state with mass balance, which implies that the predicted intake values were due to a constant rate of exposure to the phthalates or phthalate alternatives detected in dust, minimizing the need for information on additional parameters, such as chemical-specific biological half-lives and timing of exposures [57].

### 2.9. Comparing Daily Intake Rates

The contribution of residential dust concentrations to the total internal phthalate and phthalate alternative dose was determined by comparing the daily intake rates computed from residential dust concentration data along with NHANES biomonitoring data.

### 2.10. Hazard Identification

The potential human health hazards associated with exposure to each chemical substance included were summarized from the California Safer Consumer Products Candidate Chemical (SCP CC) list [58], EPA’s Integrated Risk Information System (IRIS) [59] and Pharos—Hazard Lists [60] in the form of a hazard matrix. The matrix serves as a reference to the associated health outcomes linked to the exposure to the phthalates and phthalate alternatives detected in residential dust. The criteria used to identify hazard traits are specific to the included lists. The SCP CC is comprised of 23 domestic and international authoritative lists and Pharos—Hazard Lists compiles human and environmental health information from 47 authoritative scientific lists and 32 restricted substance lists. Finally, EPA’s IRIS assessments include toxicity information used by state and local health agencies, other federal agencies, and international health organizations. The hazard traits associated with each phthalate and phthalate alternative were grouped in the following broad categories: carcinogenicity, developmental toxicity, digestive/hepatotoxicity, endocrine toxicity, eye irritation/corrosivity, immunotoxicity, neurotoxicity, ocular, skin irritation/corrosivity, reproductive toxicity, and respiratory toxicity. The strength of evidence used to identify a hazard trait was indicated in the matrix.

## 3. Results and Discussion

### 3.1. Systematic Literature Search

**Figure 1:**
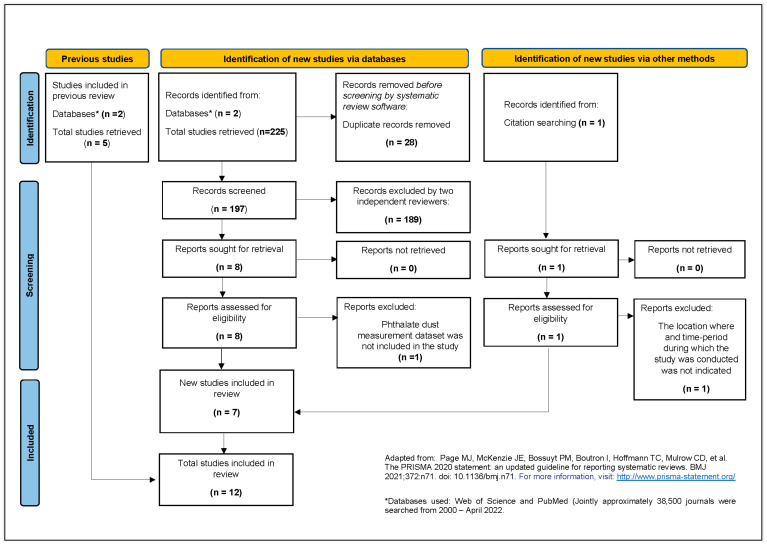
PRISMA 2020 flow chart of updated literature search and screening steps. “N” indicates the number of studies included at each step. Adapted from [61].

Our search identified 225 articles for the updated timeframe (March 2015–April 2022), of which 197 titles and abstracts were screened for relevancy. Eight were selected for full text review, of which seven were deemed relevant to our research question. The seven newly identified studies along with the five previously identified studies were included in our review for a total of 12 studies (see Figure 1). 

### 3.2. Meta-Summary of Included Studies/Strength of Evidence Assessment

From the 12 studies included in this review, 23 phthalates and phthalate alternatives were measured (see SI: Tables S1 and S2). We relied on the full reported chemical name to ensure that each phthalate and phthalate alternative was correctly identified. A little over half (*n* = 12) of these chemicals were detected with a frequency of ≥50% in the respective dust samples and were measured in at least two studies (see Table 2). Most of the studies included targeted analysis approaches, while a few were identified using suspect screening or non-target approaches [12,22,26,32,62,63,64,65,66,67,68,69].

The underlying study populations and dust samples were drawn from nine states, mostly located on the East Coast near research institutions (see SI: Figure S1). In the results of the meta-analysis, there was a lack of representation of many population subgroups, including Native and Indigenous communities that inhabit the mid-west or western US, Hawaiian and Alaskan communities. Further, the studies rarely reported extensive demographic information for individuals occupying the residential environments, making it difficult to determine the demographic groups represented in past studies. This limits the generalizability of this study, as previous studies have indicated that phthalate exposure is influenced by socioeconomic status, race/ethnicity and gender [12,20,70,71]. As such, seven of the 12 studies received a deficient ranking in the participant/house selection risk of bias domain.

We found that dust samples were collected at various temporal points from several types of residential indoor environments, such as dorms, homes and apartments. Most of the studies (*n* = 10) vacuumed shared areas in the home, such as main living areas, and provided a detailed report of the sampling techniques used. The temperature at which dust samples were stored after each home visit varied across the studies (−4 to −21 °C) and different sieve sizes (105–2000 µm) were used to sift the dust samples collected. Despite the differences in quality assurance and control (QA/QC) methods and sampling techniques used across studies, most authors provided a detailed description of their QA/QC methods and validation approaches and were given a good/adequate rating in the exposure measurement domain in the risk of bias analysis. Of the 12 studies, only two used existing vacuum bags for dust sampling, and accordingly received a deficient ranking in the exposure measurements’ risk of bias domain. 

Similarly, the treatment of data during analysis also differed across studies, but the statistical results across the studies were well-presented with detailed explanations of how missing data were addressed and the approaches taken to treat below the limit of detection values. Consequently, all the studies were given a rating of good/adequate in the analysis domain of the risk of bias analysis. Overall, 2 of the 12 studies were determined to be at risk of high bias, while the remaining 10 studies were determined to be at a low risk of bias (see SI: Figure S2).

### 3.3. Meta-Analysis of Phthalate and Phthalate Alternative Dust Concentrations

**Figure 2:**
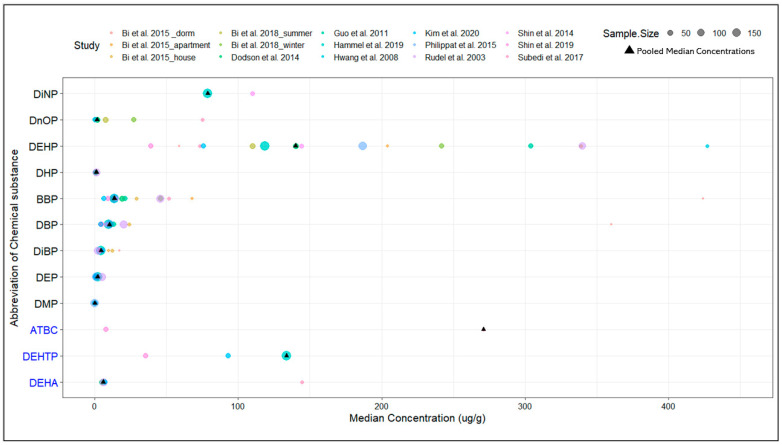
Median dust concentrations of phthalates and phthalate alternatives reported by each included study [12,22,26,32,62,63,64,65,66,67,68,69]. Circular point size is scaled to the relative sample size, and colors correspond to different studies. The triangle represents the estimated weighted and pooled median dust concentration. The abbreviation of each phthalate is shown in black, and the abbreviation of each phthalate alternative is shown in blue.

The detection frequencies and median dust concentrations recorded varied significantly among the selected studies. However, the variation in median dust concentrations of phthalates and phthalate alternatives observed in indoor dust is consistent with the variation observed in dust concentration levels for dust samples previously reported [12,22,62,69]. For phthalates, the largest variation was observed for the median dust concentrations reported for DEHP and BBP. For the phthalate alternatives, the median dust concentration levels reported for ATBC were the most variable. Notably, more studies reported data sets containing dust concentrations for phthalates compared to phthalate alternatives, with DEHP being the most reported (*n* = 15). DEHA was the most reported phthalate alternative (*n* = 4). The difference in reporting frequency may be a result of phthalates being more frequently targeted during the analysis of dust samples (see Figure 2). With less being known about which phthalate alternatives are being phased in to replace phthalates linked to adverse health outcomes, it is less likely that these compounds are included in targeted dust analyses. 

Overall, ATBC (a phthalate alternative) had the highest median concentration (271 µg/g) in indoor residential dust. Of the phthalates, the highest median concentration was observed for DEHP (140 µg/g), which had a similar median dust concentration to its structural isomer, a phthalate alternative, DEHTP (133.65 µg/g). DMP had the lowest calculated median dust concentration overall (0.07 µg/g), likely due its high volatility. The dust concentrations of phthalates and phthalate alternatives observed provide vital insights into the expected exposure profiles of individuals living in the US.

The reported median concentrations of six chemical substances (DBP, DEHA, DiBP, DEP, DnOP and DHP) in this updated study fell within the 95% confidence intervals of the geometric means reported in Mitro et al. in 2015. Two phthalates (BBP and DEHP) had lower median concentrations (13.64 and 140.00 µg/g, respectively) than the previously reported lower boundary of the reported 95% confidence interval (22.07 and 168.03 µg/g, respectively). This is likely linked to the ban of children’s toys or childcare articles containing concentrations of more than 0.1% of BBP and DEHP under the Consumer Product Safety Improvement Act (CPSIA), effective in 2008 [72]. It has been suggested that there is a rise in DEHTP and ATBC to replace phthalates, such as DEHP and BBP, that no longer comply with certain chemical regulations [11,73,74]. DEHTP and ATBC dust concentration levels were not available in Mitro et al. for comparison.

### 3.4. Sensitivity Analysis Exploring the Impact of High-Risk Bias Studies

No difference (percent point difference: <10%) was observed between the complete and low risk of bias estimates generated for all chemical substances, with the exception of DnOP (see SI: Table S5). Despite the relative percent point change observed in the estimated median dust concentration levels of DnOP, with the knowledge that no single study measured only DnOP, it is more likely that this measurement is “true”, and not one influenced by bias. Thus, the pooled weighted median dust concentrations obtained from the use of data extracted from all studies were used in the meta-analysis.

### 3.5. Daily Intake Rate Calculated from Dust Concentrations

Of the three exposure routes investigated (dust ingestion, dermal absorption from air and inhalation), dust ingestion contributed the greatest to the overall daily intake rate estimated for each high-molecular weight (HMW) phthalate and phthalate alternative (DEHP, DnOP, DINP, DEHA, ATBC and DEHTP). For low-molecular weight (LMW) phthalates (DMP, DEP, DBP, DIBP and BBP), while dust ingestion made a minute contribution to the overall daily intake, the contributions of the inhalation and dermal absorption from air routes were more significant. Notably, for the more volatile LMW compounds (DMP and DEP), dermal absorption from the air dominated the overall proportion of the intake, while inhalation accounted for about 10% of the total intake rate (see SI: Figure S4). Generally, these findings are similar to the findings of Bekö et al. [19] and further confirm that the inhalation and dermal absorption from air exposure routes are more significant for LMW compounds, while dust ingestion is a more prominent exposure route for HMW compounds.

**Figure 3:**
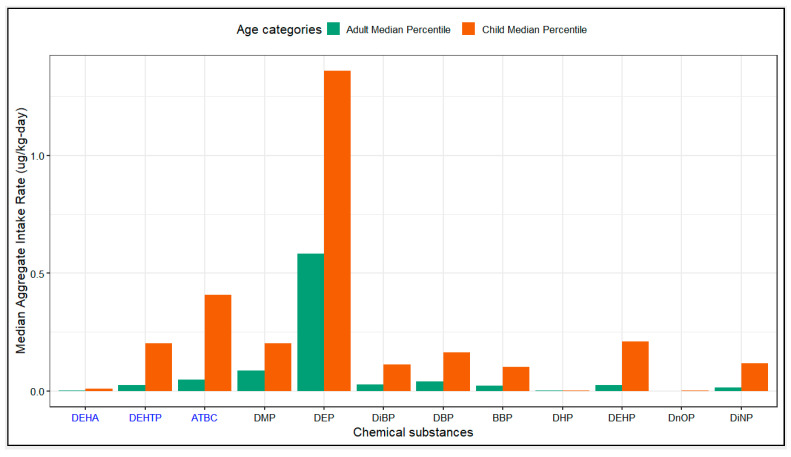
Median aggregate daily intake rates for phthalates and phthalate alternatives for children (3–5 years old) and adults (≥21 years old). On the x-axis, the abbreviation of each phthalate is in black while the abbreviation of each phthalate alternative is in blue. Within each grouping, the chemicals are arranged in order of increasing molecular mass.

The aggregate daily intake rates estimated from dust concentrations for all phthalates and phthalate alternatives were more than two-times higher for children than adults (see Figure 3). This is as expected, as children breathe and eat more per unit mass, have a larger body surface area to mass ratio than adults, and tend exhibit behaviors that increase their exposure to dust such as crawling and “mouthing” objects found around the home. DEP had the highest aggregate daily intake rate (1.36 ug/kg-day for children) despite having the lowest concentration in dust, followed by ATBC, DMP, DEHP and DEHTP. The daily intake rate for DnOP and DHP, and the phthalate alternative, DEHA, were very low in comparison to other measured phthalates or phthalate alternatives.

### 3.6. Estimated Urine Metabolite Concentration of Phthalates and Phthalate Alternatives

Of the 12 phthalates and phthalate alternatives included in the meta-analysis, only 7 had metabolites that were detected in urine and reported in the NHANES 2017–2018 cohort. We mapped the phthalates and phthalate alternative metabolites to their parent molecules using the information provided in Center of Disease Control’s Laboratory Procedure Manual associated with that cohort group [75] (see SI: Figure S5).

**Figure 4:**
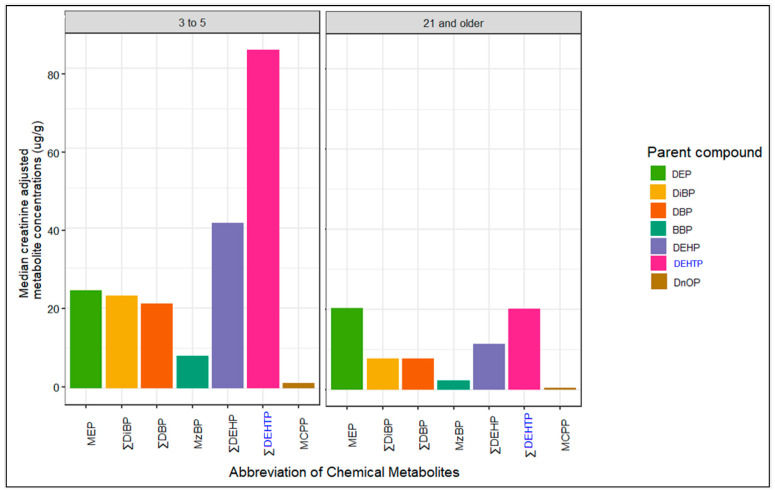
Median creatinine-adjusted phthalate and phthalate alternative metabolite concentrations (ug/g) from NHANES 2017–2018 survey year for children (3–5 years) and adults (21 years and older). Chemicals are arranged in order of increasing molecular mass. On the x-axis, the abbreviation of each phthalate is in black while the abbreviation of each phthalate alternative is in blue.

The sum of the metabolites of the phthalate alternative DEHTP (ΣDEHTP) had the highest adjusted urinary creatinine concentration for children (3–5 years old), followed by the sum of the metabolites for DEHP (ΣDEHP) and the metabolite of DEP (MEP) (see Figure 4). For adults, MEP and ΣDEHTP had the highest urinary creatinine-adjusted concentrations, followed by ΣDEHP. These metabolite concentrations indicate that it is likely that exposure to sources containing DEHTP, DEP and DEHP is more common in the US population for these age groups. DEHTP is commonly used in children’s toys and food contact materials as a replacement for DEHP [76], while DEP is commonly found in cosmetics and other fragranced products due to its ability to help scents last longer [4]. Despite children generally having higher exposure levels than adults to the examined chemical substances, the urinary concentration of MEP was similar for both children and adults. This indicates that exposure to products containing DEP, such as cosmetics, medications, lotions and hair-cleaning products, might be similar for adults and children.

### 3.7. Daily Intake Rates from Dust Compared to Daily Intake Rates from NHANES

**Figure 5:**
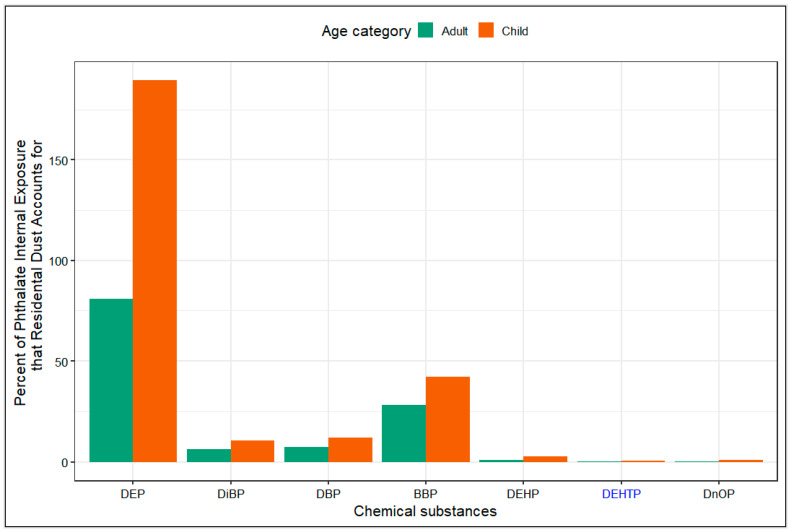
Percentage contribution of dust daily intake rate to overall daily intake rate of phthalates and phthalate alternatives for children and adults in the US general population. Chemicals are arranged in order of increasing molecular mass. On the x-axis, the abbreviation of each phthalate is in black while the abbreviation of each phthalate alternative is in blue.

Intake rates obtained from indoor dust concentrations were compared to intake rates obtained from NHANES 2017–2018 cycle biomonitoring data. Dust accounted for most of DEP exposure in both age groups investigated (see Figure 5). In adults, 81% of the total predicted intake rate of DEP was found to be attributed to exposure to DEP concentrations in dust from the three exposure routes investigated. While in children, the predicted DEP dust intake rate exceeded the predicted total DEP intake rate (189%). A similar pattern of exceedance of total DEP intake estimated from urinary metabolite levels have been previously observed [19,67]. This exceedance may be linked to the variability in urinary MEP levels expected throughout the day, as the use of personal care products containing DEP varies widely and temporally. This exceedance may also be linked to the limited reliability of gas-phase concentration predictions for DEP. Dust also accounted for a measurable proportion of BBP, DiBP and DBP total internal exposure for adults and children (see Figure 5). For the phthalate alternative, DEHTP, which has elevated dust concentrations and high internal exposure levels in children and adults [11,73,74], dust contributed negligibly to the total internal dose (<5%). The contribution of dust to the total internal dose of DEHP, a phthalate with significant indoor dust concentrations and internal exposure levels, was also very small (<5%).

Despite the increasing concentrations of several phthalate alternatives observed in the indoor dust exposure profile, during the 2017–2018 NHANES survey cycle, only the metabolites of the phthalate alternative, DEHTP, were measured in urine. Although ATBC had the highest median concentration in dust and one of the highest daily intake rates for both age groups (see Figure 2 and Figure 3), the metabolites of ATBC were not measured in the NHANES 2017–2018 cycle, so we were not able to estimate its contribution to internal exposure levels.

### 3.8. Overview of Hazard Identification

**Figure 6:**
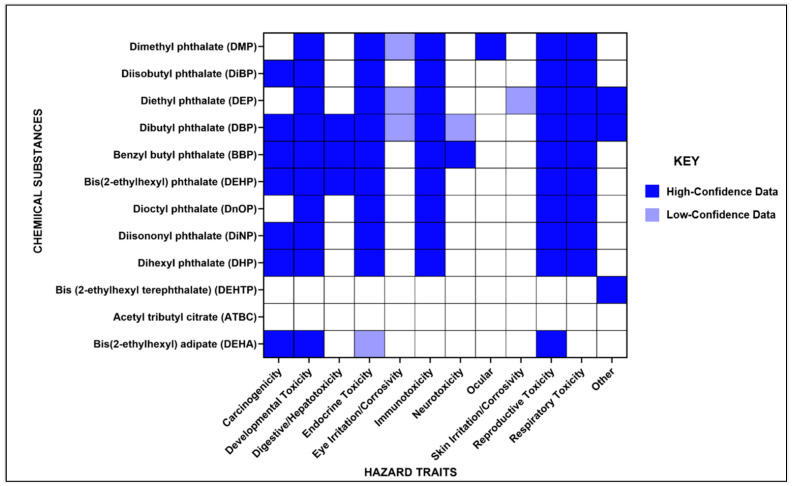
Hazard traits compiled from IRIS, SCP CCL and Pharos that are associated with phthalates and phthalate alternatives, detected in indoor dust and included in meta-analysis. In the matrix, hazard traits supported by high-confidence data as described by each source are indicated in dark blue while traits supported by low-confidence data are indicated in purple.

Human health hazard traits associated with the phthalates and phthalate alternatives included in our analysis were identified using IRIS, SCP CCL and Pharos (Figure 6). The hazard information indicated in the matrix in Figure 6 is not exhaustive and should not be considered a complete representation of the human health hazards associated with the chemicals of concern. Of the hazard traits included, carcinogenicity, developmental, endocrine, reproductive, and respiratory toxicity were most commonly associated with the phthalates included in our analysis. Multiple phthalates shared common adverse health outcomes, including five phthalates that are currently undergoing TSCA risk evaluation (BBP, DBP, DEHP, DiBP and DiNP). For four of these phthalates, BBP, DBP, DiBP and DEHP, dust contributed 42%, 12%, 11% and 3%, respectively, of the total internal exposure observed in children (3 to 5 years old) and 28%, 7%, 7% and 1%, respectively, of the total internal exposure observed in adults (≥21 years).

Overall, less hazard information was available for phthalate alternatives compared to phthalates. The phthalate alternatives detected at higher levels in the indoor environment and in biomonitoring data (ATBC and DEHTP) are currently not linked to any of the major hazard traits included in our hazard matrix, which is possibly due to the limited availability of toxicity data. 

## 4. Limitations of the Study

This systematic review and meta-analysis identified only 12 studies which measured phthalates and/or phthalate alternatives in the indoor residential environment. Additionally, the underlying data extracted from the included studies represented only nine states primarily located on the East Coast, thereby limiting the generalizability of the results. Further, due to the difficulty with obtaining exposure data for all possible exposure scenarios, we relied on modeled data and reported physiochemical parameters to estimate the contribution of indoor dust exposures to the total internal dose of phthalates and phthalate alternatives [36,77,78]. However, due to the inherent uncertainties associated with modeled data, the point estimates we relied on were expected to vary within the margin of error associated with each prediction. 

Further, our predicted distribution of phthalate and phthalate alternative exposure in the US general population from dust captured only the exposures expected at the 50th percentile, thereby failing to capture the exposures occurring in highly-exposed and susceptible subgroups, including certain populations that face a greater risk of experiencing adverse health effects from indoor chemical exposures when compared to the median population [5,79,80]. In addition, indoor chemical exposure disparities have been linked to certain sociodemographic indicators and non-chemical stressors, such as dilapidated housing, poor sanitation, and differing behavioral and consumption patterns linked to race and socio-economic class [79,80]. However, it was difficult to examine potential associations between these factors and indoor dust phthalate and phthalate alternative exposures for individuals in study households, as sociodemographic information was poorly recorded across studies. Even with the numerous calls in the literature for exposure studies focused primarily on the detection of chemicals in the indoor environment, in order to incorporate questionnaire data so that demographic characteristics of residents are better captured, this remains a significant gap in the literature. It therefore remains a challenge to characterize phthalate and phthalate alternative exposure patterns across demographic factors in the US population.

Despite the aforementioned limitations, this study provides novel insights into the contributions of dust-related exposure sources and routes to a person’s total phthalate and phthalate alternative exposure in the US. In addition, this study highlights a critical data gap for phthalate alternatives being measured in the indoor environment and the corresponding biomarkers of exposure being measured in individuals. As phthalate alternative levels, such as ATBC, continue to rise in the indoor environment, researchers should seek to measure the dust and urinary metabolite concentrations of these chemical substances. Further, this study adds to a growing body of evidence that measurements of chemical substances in dust can reliably predict human exposure and support phthalate and phthalate alternative biomonitoring efforts. 

## 5. Conclusions

Indoor dust can significantly contribute to daily intake levels of phthalates and phthalate alternatives via ingestion, inhalation, and dermal absorption from air, and has a greater influence on the total internal dose of these chemicals in children compared to adults. For the phthalates BBP, DBP, DiBP, and DEHP that are currently undergoing a risk evaluation under TSCA, we observed that dust contributed to 42%, 12%, 11% and 3%, respectively, of the total internal exposure observed in children (3 to 5 years old) and 28%, 7%, 7% and 1%, respectively, of the total internal exposure observed in adults (≥21 years). Further, we observed that the relative contribution of dust to the overall internal dose of phthalates and phthalate alternatives varied according to the physio-chemical characteristics of the chemical. Dust contributed more significantly to the overall intake of LMW chemicals, such as DEP and BBP, which are more likely to partition to the indoor air from dust. In comparison, the contribution of dust as a source to the overall internal dose of HMW phthalates, such as DEHTP and DEHP, was minimal. This illustrates the importance of understanding the molecular exchange of chemicals between the phases and reservoirs in the indoor environment when estimating indoor exposure for volatile or semi-volatile chemical substances. 

This study highlights the increased need for researchers to measure phthalate alternatives levels in indoor environment matrices and in serum, as data availability in the literature was limited. In addition, there is a need for exposure studies investigating the concentrations of chemical substances in the indoor environment to report demographic information. Future studies should seek to characterize the contribution of dust to a total internal phthalate and phthalate alternative dose for individuals who have disproportionately high exposures or are more susceptible to harm from chemical exposures, and the relative contribution of dust to tolerable daily intake levels across exposure percentiles. 

## Figures and Tables

**Table 1:** Exposure Factors for Both Adults (≥21 years old) and Children (3–<6 years), and Equations Used to Estimate Intake Rate from Dust, Dermal Uptake from the Gas Phase, and Inhalation of Air.

Dust Ingestion Intake, ug/kg-day = (Dust Conc. xIR × ED)/BW		
*Exposure Factors*	*Value*	*Source*
Dust Concentration data (Conc.): ug/g dust	Study data (see Table S11)	-
Ingestion Rate (IR): g dust/day	Child (2 to <6 years) ^2^: 0.030 (central tendency) 0.100 (95th percentile)	US EPA Exposure Factors Handbook Table 5-1 (2017 update)
	Adult: 0.020 (central tendency) 0.060 (95th percentile)	US EPA Exposure Factors Handbook Table 5-1 (2017 update)
Exposure duration at home (fraction of time per day spent at home)	Child: 0.861	Abdallah et al. (2014) [34]
	Adult: 0.638	Abdallah et al. (2014) [34]
Body Weight (BW): kg	Child (3 to <6 years) ^2^: 17.8 (central tendency)	US EPA Exposure Factors Handbook Table 8.3 (2011)
	Adult: 76.9 (central tendency)	US EPA Exposure Factors Handbook Table 8.3 (2011)
**Air Inhalation Intake, ug/day-kg = (Total Air Conc. (ug/m^3^) × Vol. of Air Inhaled × ED)/BW**		
Total Air Concentration (ug/m^3^)	Varies by chemical ^1^	-
Volume of Air Inhaled (m^3^/day)	Child (3 to <6 years) ^2^: 12.64 (central tendency) 15.41 (95th percentile)	US EPA Exposure Factors Handbook Table 6-16 (2011)
	Adult: 18.63 (central tendency) 25.16 (95th percentile)	US EPA Exposure Factors Handbook Table 6-16 (2011)
**Dermal Exposure through Air Intake, ug/kg-day = (Gaseous Air Conc. (ug/m^3^) × (Indoor Air Transdermal Permeability /100) × Body SA × ET)/BW**		
Gaseous Air Concentration (ug/m^3^)	Varies by chemical ^1^	-
Indoor Air Transdermal Permeability (cm)	Varies by chemical ^1^	-
Body Surface Area, m^2^	Child (3 to <6 years): 0.76 (central tendency) 0.95 (95th percentile)	US EPA Exposure Factors Handbook Table 7-1 (2011)
	Adult (male) ^3^: 2.07 (central tendency) 2.47 (95th percentile)	US EPA Exposure Factors Handbook Table 7-1 (2011)
Exposure in hours (daily)	Child: 21	Based on assumed exposure duration at home, observed above
	Adult: 15	Based on assumed exposure duration at home, observed above

(1) See Supplemental Information for the equations used to calculate these parameters. (2) The recommended values for dust ingestion, body weight, and volume of air inhaled were averaged across the relevant age groups, as referenced in the indicated version of the EPA Exposure Factors Handbook. Adults were defined as individuals 21 years and older unless otherwise defined in the Exposure Factors Handbook. (3) The recommended values for total body surface area for adult males (averaged) was used as the surface area. This is because males on average have a larger surface area than females.

**Table 2:** Phthalates and Phthalate Alternatives Included in Meta-Analysis.

CASRN	Abbreviation	Substance Name
131-11-3	DMP	Dimethyl phthalate
84-69-5	DiBP	Diisobutyl phthalate
84-66-2	DEP	Diethyl phthalate
84-74-2	DBP	Dibutyl phthalate
85-68-7	BBP	Benzyl butyl phthalate
117-81-7	DEHP	Bis(2-ethylhexyl) phthalate
84-75-3	DHP	Dihexyl phthalate
117-84-0	DnOP	Dioctyl phthalate
28553-12-0	DiNP	Diisononyl phthalate
4654-26-6; 6422-86-2	DOTP, DEHT or DEHTP	Dioctyl terephthalate or bis (2-ethylhexyl terephthalate)
77-90-7	ATBC	Acetyl tributyl citrate
103-23-1	DEHA	Bis(2-ethylhexyl) adipate or di (2-ethylhexyl) adipate

## Data Availability

No new data were generated. All data resources are publicly available.

## Supplementary Materials

The following supporting information can be downloaded at: https://www.mdpi.com/article/10.3390/ijerph20166589/s1, Table S1: List of ortho-phthalates and ortho-phthalate alternatives that are commonly detected (frequency ≥ 50) in the included studies, Table S2: List of ortho-phthalates and ortho-phthalate alternatives that are commonly detected (frequency < 50) in the included studies, Table S3: Systematic Review Search Strategy (for the updated review), Table S4: References of Studies Excluded At Full Study Stage in Systematic Review, Figure S1: Geographic Regions Represented by Studies Included in Meta-Analysis, Figure S2: Risk of bias ratings in exposure measurement, participant/house selection and analysis domains, Table S5: Sensitivity analysis exploring the influence of bias on the pooled median chemical concentrations, Table S6: Qualitative Data Extraction and Summary, Table S7: Quality assurance/quality control measures, Table S8: Treatment of below-method of detection limit (MDL) values, Table S9: Measure of central tendency and measure of spread reported, Table S10: Internal risk of bias assessment for studies included in meta-analysis, Table S11: The weighted pooled median of ortho-phthalates and ortho-phthalate alternatives that were commonly detected (frequency ≥ 50) in at least two studies, Figure S3: Weighted pooled median phthalate and phthalate alternative concentrations in the indoor residential environment across sub-populations in the United States, Figure S4: Proportion of intake for children (3 to <6 years old) across three exposure pathways: ingestion, air inhalation, and dermal from air, Equations used for modelling intake rate from dust, Table S12: Urine metabolites of detected phthalates and phthalate alternatives, Figure S5: Parent-metabolite mapping for phthalate and phthalate alternative metabolites of chemical substances identified in systematic review and measured in urine during the NHANES 2017–2018 cycle, Table S13: Excretion factors used to estimate daily intake from phthalate and phthalate alternative urinary metabolites, Table S14: Summary of data obtained from NHANES 2017–2018 Survey Year, Figure S6: Correlation plot between molecular weight of phthalates and phthalates alternatives and their relative contribution to internal phthalate dose in the US population.

## References

[1] Phthalates Factsheet|National Biomonitoring Program|CDC. https://www.cdc.gov/biomonitoring/Phthalates_FactSheet.html.

[2] Kashyap D., Agarwal T. (2018). Concentration and Factors Affecting the Distribution of Phthalates in the Air and Dust: A Global Scenario. Sci. Total Environ..

[3] Langer S., Fredricsson M., Weschler C., Beko G., Strandberg B., Remberger M., Toftum J., Clausen G. (2016). Organophosphate Esters in Dust Samples Collected from Danish Homes and Daycare Centers. Chemosphere.

[4] EPA (2017). America’s Children and the Environment Phthalates: Biomonitoring, Third Edition. https://www.epa.gov/sites/default/files/2017-08/documents/phthalates_updates_live_file_508_0.pdf.

[5] Mitro S., Dodson R., Singla V., Adarnkiewicz G., Elmi A., Tilly M., Zota A. (2016). Consumer Product Chemicals in Indoor Dust: A Quantitative Meta-Analysis of US Studies. Environ. Sci. Technol..

[6] Zota A.R., Singla V., Adamkiewicz G., Mitro S.D., Dodson R.E. (2017). Reducing Chemical Exposures at Home: Opportunities for Action. J. Epidemiol. Community Health.

[7] Jurewicz J., Hanke W. (2011). Exposure to Phthalates: Reproductive Outcome and Children Health. A Review of Epidemiological Studies. Int. J. Occup. Med. Environ. Health.

[8] Engel S., Patisaul H., Brody C., Hauser R., Zota A., Bennet D., Swanson M., Whyatt R. (2021). Neurotoxicity of Ortho-Phthalates: Recommendations for Critical Policy Reforms to Protect Brain Development in Children. Am. J. Public. Health.

[9] CPSC Prohibits Certain Phthalates in Children’s Toys and Child Care Products. https://www.cpsc.gov/Newsroom/News-Releases/2018/CPSC-Prohibits-Certain-Phthalates-in-Childrens-Toys-and-Child-Care-Products.

[10] Qadeer A., Kirsten K.L., Ajmal Z., Jiang X., Zhao X. (2022). Alternative Plasticizers as Emerging Global Environmental and Health Threat: Another Regrettable Substitution?. Environ. Sci. Technol..

[11] Bui T.T., Giovanoulis G., Cousins A.P., Magnér J., Cousins I.T., de Wit C.A. (2016). Human Exposure, Hazard and Risk of Alternative Plasticizers to Phthalate Esters. Sci. Total Environ..

[12] Hammel S.C., Levasseur J.L., Hoffman K., Phillips A.L., Lorenzo A.M., Calafat A.M., Webster T.F., Stapleton H.M. (2019). Children’s Exposure to Phthalates and Non-Phthalate Plasticizers in the Home: The TESIE Study. Environ. Int..

[13] Wormuth M., Scheringer M., Vollenweider M., Hungerbühler K. (2006). What Are the Sources of Exposure to Eight Frequently Used Phthalic Acid Esters in Europeans?. Risk Anal..

[14] Fromme H., Bolte G., Koch H.M., Angerer J., Boehmer S., Drexler H., Mayer R., Liebl B. (2007). Occurrence and Daily Variation of Phthalate Metabolites in the Urine of an Adult Population. Int. J. Hyg. Environ. Health.

[15] Adibi J.J., Perera F.P., Jedrychowski W., Camann D.E., Barr D., Jacek R., Whyatt R.M. (2003). Prenatal Exposures to Phthalates among Women in New York City and Krakow, Poland. Environ. Health Perspect..

[16] Franco A., Prevedouros K., Alli R., Cousins I.T. (2007). Comparison and Analysis of Different Approaches for Estimating the Human Exposure to Phthalate Esters. Environ. Int..

[17] US EPA, O Chemicals Undergoing Risk Evaluation under TSCA. https://www.epa.gov/assessing-and-managing-chemicals-under-tsca/chemicals-undergoing-risk-evaluation-under-tsca.

[18] Diamond M.L., Okeme J.O., Melymuk L. (2021). Hands as Agents of Chemical Transport in the Indoor Environment. Environ. Sci. Technol. Lett..

[19] Bekö G., Weschler C.J., Langer S., Callesen M., Toftum J., Clausen G. (2013). Children’s Phthalate Intakes and Resultant Cumulative Exposures Estimated from Urine Compared with Estimates from Dust Ingestion, Inhalation and Dermal Absorption in Their Homes and Daycare Centers. PLoS ONE.

[20] Branch F., Woodruff T.J., Mitro S.D., Zota A.R. (2015). Vaginal Douching and Racial/Ethnic Disparities in Phthalates Exposures among Reproductive-Aged Women: National Health and Nutrition Examination Survey 2001–2004. Environ. Health.

[21] National Research Council (US) Committee on the Health Risks of Phthalates (2008). Phthalates and Cumulative Risk Assessment: The Tasks Ahead.

[22] Bi X., Yuan S., Pan X., Winstead C., Wang Q. (2015). Comparison, Association, and Risk Assessment of Phthalates in Floor Dust at Different Indoor Environments in Delaware, USA. J. Environ. Sci. Health A Tox. Hazard. Subst. Environ. Eng..

[23] Not Just Dirt: Toxic Chemicals in Indoor Dust. https://www.nrdc.org/resources/not-just-dirt-toxic-chemicals-indoor-dust.

[24] Butte W., Heinzow B. (2002). Pollutants in House Dust as Indicators of Indoor Contamination. Rev. Environ. Contam. Toxicol..

[25] Weschler C.J., Nazaroff W.W. (2010). SVOC Partitioning between the Gas Phase and Settled Dust Indoors. Atmos. Environ..

[26] Dodson R.E., Camann D.E., Morello-Frosch R., Brody J.G., Rudel R.A. (2015). Semivolatile Organic Compounds in Homes: Strategies for Efficient and Systematic Exposure Measurement Based on Empirical and Theoretical Factors. Environ. Sci. Technol..

[27] Little J.C., Weschler C.J., Nazaroff W.W., Liu Z., Cohen Hubal E.A. (2012). Rapid Methods to Estimate Potential Exposure to Semivolatile Organic Compounds in the Indoor Environment. Environ. Sci. Technol..

[28] Weschler C.J., Nazaroff W.W. (2012). SVOC Exposure Indoors: Fresh Look at Dermal Pathways. Indoor Air.

[29] US EPA O. Understanding Exposures in Children’s Environments. https://www.epa.gov/healthresearch/understanding-exposures-childrens-environments.

[30] Covidence Systematic Review Software, Veritas Health Innovation, Melbourne, Australia. www.Covidence.org.

[31] DeLuca N.M., Angrish M., Wilkins A., Thayer K., Cohen Hubal E.A. (2021). Human Exposure Pathways to Poly- and Perfluoroalkyl Substances (PFAS) from Indoor Media: A Systematic Review Protocol. Environ. Int..

[32] Bi C., Maestre J., Li H., Zhang G., Givehchi R., Mahdavi A., Kinney K., Siegel J., Horner S., Xu Y. (2018). Phthalates and Organophosphates in Settled Dust and HVAC Filter Dust of US Low-Income Homes: Association with Season, Building Characteristics, and Childhood Asthma. Environ. Int..

[33] McGrath S., Zhao X., Qin Z.Z., Steele R., Benedetti A. (2019). One-Sample Aggregate Data Meta-Analysis of Medians. Stat. Med..

[34] Abdallah M.A.-E., Covaci A. (2014). Organophosphate Flame Retardants in Indoor Dust from Egypt: Implications for Human Exposure. Environ. Sci. Technol..

[35] Li R., Kang L., Wu S., Zhou X., Wang X. (2023). Effect of Dust Formation on the Fate of Indoor Phthalates: Model Analysis. Build. Environ..

[36] Schossler P., Schripp T., Salthammer T., Bahadir M. (2011). Beyond Phthalates: Gas Phase Concentrations and Modeled Gas/Particle Distribution of Modern Plasticizers. Sci. Total Environ..

[37] Williams A.J., Grulke C.M., Edwards J., McEachran A.D., Mansouri K., Baker N.C., Patlewicz G., Shah I., Wambaugh J.F., Judson R.S. (2017). The CompTox Chemistry Dashboard: A Community Data Resource for Environmental Chemistry. J. Cheminform..

[38] European Chemical Agency European Chemical Agency Assessment of Regulatory Needs: Ortho-Phthalates 2021. https://echa.europa.eu/documents/10162/7adf54e4-22de-fbbf-8b79-a709681d6f4a.

[39] European Chemical Agency (2021). European Chemical Agency Assessment of Regulatory Needs: Isophthalates, Terephthalates and Trimellitates. https://echa.europa.eu/documents/10162/c2bd91c0-1593-1ff3-1551-009204638501.

[40] NHANES Questionnaires, Datasets, and Related Documentation. https://wwwn.cdc.gov/nchs/nhanes/Default.aspx.

[41] Saravanabhavan G., Walker M., Guay M., Aylward L. (2014). Urinary Excretion and Daily Intake Rates of Diethyl Phthalate in the General Canadian Population. Sci. Total Environ..

[42] Huang P.-C., Cheng P.-K., Chen H.-C., Shiue I., Chang W.-T., Huang H.-I., Chang J.-W., Wang I.-J. (2022). Are Phthalate Exposure Related to Oxidative Stress in Children and Adolescents with Asthma? A Cumulative Risk Assessment Approach. Antioxidants.

[43] David R.M. (2000). Exposure to Phthalate Esters. Environ. Health Perspect..

[44] Koch H.M., Drexler H., Angerer J. (2003). An Estimation of the Daily Intake of Di(2-Ethylhexyl)Phthalate (DEHP) and Other Phthalates in the General Population. Int. J. Hyg. Environ. Health.

[45] Chang J.-W., Lee C.-C., Pan W.-H., Chou W.-C., Huang H.-B., Chiang H.-C., Huang P.-C. (2017). Estimated Daily Intake and Cumulative Risk Assessment of Phthalates in the General Taiwanese after the 2011 DEHP Food Scandal. Sci. Rep..

[46] Wang B., Wang H., Zhou W., Chen Y., Zhou Y., Jiang Q. (2015). Urinary Excretion of Phthalate Metabolites in School Children of China: Implication for Cumulative Risk Assessment of Phthalate Exposure. Environ. Sci. Technol..

[47] Lumley T. (2004). Analysis of Complex Survey Samples. J. Stat. Softw..

[48] Mage D.T., Allen R.H., Gondy G., Smith W., Barr D.B., Needham L.L. (2004). Estimating Pesticide Dose from Urinary Pesticide Concentration Data by Creatinine Correction in the Third National Health and Nutrition Examination Survey (NHANES-III). J. Expo. Anal. Environ. Epidemiol..

[49] Mage D.T., Allen R.H., Kodali A. (2008). Creatinine Corrections for Estimating Children’s and Adult’s Pesticide Intake Doses in Equilibrium with Urinary Pesticide and Creatinine Concentrations. J. Expo. Sci. Environ. Epidemiol..

[50] Organophosphate Metabolites in Urine Samples from Danish Children and Women. https://mst.dk/service/publikationer/publikationsarkiv/2016/sep/democrophes/.

[51] Lee I., Pälmke C., Ringbeck B., Ihn Y., Gotthardt A., Lee G., Alakeel R., Alrashed M., Tosepu R., Jayadipraja E.A. (2021). Urinary Concentrations of Major Phthalate and Alternative Plasticizer Metabolites in Children of Thailand, Indonesia, and Saudi Arabia, and Associated Risks. Environ. Sci. Technol..

[52] Anderson W.A.C., Castle L., Hird S., Jeffery J., Scotter M.J. (2011). A Twenty-Volunteer Study Using Deuterium Labelling to Determine the Kinetics and Fractional Excretion of Primary and Secondary Urinary Metabolites of Di-2-Ethylhexylphthalate and Di-Iso-Nonylphthalate. Food Chem. Toxicol..

[53] Anderson W.A., Castle L., Scotter M.J., Massey R.C., Springall C. (2001). A Biomarker Approach to Measuring Human Dietary Exposure to Certain Phthalate Diesters. Food Addit. Contam..

[54] Itoh H., Yoshida K., Masunaga S. (2007). Quantitative Identification of Unknown Exposure Pathways of Phthalates Based on Measuring Their Metabolites in Human Urine. Environ. Sci. Technol..

[55] Koch H.M., Wittassek M., Brüning T., Angerer J., Heudorf U. (2011). Exposure to Phthalates in 5-6 Years Old Primary School Starters in Germany--a Human Biomonitoring Study and a Cumulative Risk Assessment. Int. J. Hyg. Environ. Health.

[56] Lessmann F., Correia-Sá L., Calhau C., Domingues V.F., Weiss T., Brüning T., Koch H.M. (2017). Exposure to the Plasticizer Di(2-Ethylhexyl) Terephthalate (DEHTP) in Portuguese Children—Urinary Metabolite Levels and Estimated Daily Intakes. Environ. Int..

[57] Stanfield Z., Setzer R.W., Hull V., Sayre R.R., Isaacs K.K., Wambaugh J.F. (2022). Bayesian Inference of Chemical Exposures from NHANES Urine Biomonitoring Data. J. Expo. Sci. Environ. Epidemiol..

[58] Home: CalSAFER. https://calsafer.dtsc.ca.gov/.

[59] US EPA, O Basic Information about the Integrated Risk Information System. https://www.epa.gov/iris/basic-information-about-integrated-risk-information-system.

[60] Pharos—About. https://pharos.healthybuilding.net/about?_gl=1*rqv3wh*_ga*MTQ1NDEzNzcyNy4xNjczOTAyMTA1*_ga_9HBMPKD00P*MTY3MzkwMjEwNC4xLjEuMTY3MzkwMjMwOS4wLjAuMA.

[61] Page M.J., McKenzie J.E., Bossuyt P.E., Boutron I., Hoffmann T.C., Mulrow C.D., Shamseer L., Tetzlaff J.M., Akl E.A., Brenna S.E. (2021). The PRISMA 2020 Statement: An Updated Guideline for Reporting Systematic Reviews. BMJ.

[62] Kim K., Shin H., Wong L., Young T., Bennett D. (2021). Temporal Variability of Indoor Dust Concentrations of Semivolatile Organic Compounds. Indoor Air.

[63] Shin H.-M., McKone T.E., Nishioka M.G., Fallin M.D., Croen L.A., Hertz-Picciotto I., Newschaffer C.J., Bennett D.H. (2014). Determining Source Strength of Semivolatile Organic Compounds Using Measured Concentrations in Indoor Dust. Indoor Air.

[64] Shin H., Moschet C., Young T., Bennett D. (2020). Measured Concentrations of Consumer Product Chemicals in California House Dust: Implications for Sources, Exposure, and Toxicity Potential. Indoor Air.

[65] Hwang H.-M., Park E.-K., Young T.M., Hammock B.D. (2008). Occurrence of Endocrine-Disrupting Chemicals in Indoor Dust. Sci. Total Environ..

[66] Rudel R.A., Camann D.E., Spengler J.D., Korn L.R., Brody J.G. (2003). Phthalates, Alkylphenols, Pesticides, Polybrominated Diphenyl Ethers, and Other Endocrine-Disrupting Compounds in Indoor Air and Dust. Environ. Sci. Technol..

[67] Guo Y., Kannan K. (2011). Comparative Assessment of Human Exposure to Phthalate Esters from House Dust in China and the United States. Environ. Sci. Technol..

[68] Subedi B., Sullivan K.D., Dhungana B. (2017). Phthalate and Non-Phthalate Plasticizers in Indoor Dust from Childcare Facilities, Salons, and Homes across the USA. Environ. Pollut..

[69] Philippat C., Bennett D., Krakowiak P., Rose M., Hwang H., Hertz-Picciotto I. (2015). Phthalate Concentrations in House Dust in Relation to Autism Spectrum Disorder and Developmental Delay in the CHildhood Autism Risks from Genetics and the Environment (CHARGE) Study. Environ. Health.

[70] Mitro S.D., Chu M.T., Dodson R.E., Adamkiewicz G., Chie L., Brown F.M., James-Todd T.M. (2019). Phthalate Metabolite Exposures among Immigrants Living in the United States: Findings from NHANES, 1999–2014. J. Expo. Sci. Environ. Epidemiol..

[71] James-Todd T., Meeker J., Huang T., Hauser R., Seely E., Ferguson K., Rich-Edwards J., McElrath T. (2017). Racial and Ethnic Variations in Phthalate Metabolite Concentrations across Pregnancy. J. Expo. Sci. Environ. Epidemiol..

[72] Phthalates Business Guidance & Small Entity Compliance Guide. https://www.cpsc.gov/Business--Manufacturing/Business-Education/Business-Guidance/Phthalates-Information.

[73] Silva M.J., Samandar E., Calafat A.M., Ye X. (2015). Identification of Di-2-Ethylhexyl Terephthalate (DEHTP) Metabolites Using Human Liver Microsomes for Biomonitoring Applications. Toxicol. Vitr..

[74] Nagorka R., Conrad A., Scheller C., Süssenbach B., Moriske H.-J. (2011). Diisononyl 1,2-Cyclohexanedicarboxylic Acid (DINCH) and Di(2-Ethylhexyl) Terephthalate (DEHT) in Indoor Dust Samples: Concentration and Analytical Problems. Int. J. Hyg. Environ. Health.

[75] Phthalates and Plasticizers Metabolites-Urine (PHTHTE_J). https://wwwn.cdc.gov/Nchs/Nhanes/2017-2018/PHTHTE_J.htm#Description_of_Laboratory_Methodology.

[76] Rodríguez-Carmona Y., Ashrap P., Calafat A.M., Ye X., Rosario Z., Bedrosian L.D., Huerta-Montanez G., Vélez-Vega C.M., Alshawabkeh A., Cordero J.F. (2020). Determinants and Characterization of Exposure to Phthalates, DEHTP and DINCH among Pregnant Women in the PROTECT Birth Cohort in Puerto Rico. J. Expo. Sci. Environ. Epidemiol..

[77] Schripp T. (2009). Distribution of Phthalates in the Indoor Environment: Application and Evaluation of Indoor Air Models. Ph.D. Thesis.

[78] Cherrie J.W., Fransman W., Heussen G.A.H., Koppisch D., Jensen K.A. (2020). Exposure Models for REACH and Occupational Safety and Health Regulations. Int. J. Environ. Res. Public Health.

[79] Adamkiewicz G., Zota A.R., Fabian M.P., Chahine T., Julien R., Spengler J.D., Levy J.I. (2011). Moving Environmental Justice Indoors: Understanding Structural Influences on Residential Exposure Patterns in Low-Income Communities. Am. J. Public. Health.

[80] Gochfeld M., Burger J. (2011). Disproportionate Exposures in Environmental Justice and Other Populations: The Importance of Outliers. Am. J. Public. Health.

