# Supplementary material for: Characterizing the Contribution of Indoor Residential Phthalate and Phthalate Alternative Dust Concentrations to Internal Dose in the US General Population: An Updated Systematic Review and Meta-Analysis
Source: Int J Environ Res Public Health. 2023 Aug 17;20(16):6589. doi: 10.3390/ijerph20166589 (PMC10454216; doi:10.3390/ijerph20166589)

Characterizing the Contribution of Indoor Residential Phthalate and Phthalate Alternative Dust Concentrations to Internal Dose in the US General Population: An Updated Systematic Review and Meta-Analysis

Sashoy G. Milton [1], Rachel A. Tejiram [2], Rashmi Joglekar [3,*] and Kate Hoffman [1,*]

[1] Nicholas School of the Environment, Duke University, Durham, NC 27710, USA
[2] Nash Family Department of Neuroscience, Icahn School of Medicine at Mount Sinai, New York, NY 10029,USA
[3] Earthjustice, Toxic Exposure and Health Program, Washington, DC 20001, USA
* Correspondence: rashmij214@gmail.com (R.J.); kate.hoffman@duke.edu (K.H.)

Table of Contents for Supplementary Information

Table S1: List of ortho-phthalates and ortho-phthalate alternatives that are commonly detected (frequency ⩾ 50) in the included studies 4

Table S2: List of *ortho*-phthalates and *ortho*-phthalate alternatives that are commonly detected (frequency < 50) in the included studies. 5

List of Abbreviations Used in Table S1 and S2 6

Table S3. Systematic Review Search Strategy (for the updated review) 6

Table S4. References of Studies Excluded At Full Study Stage in Systematic Review: 7

Figure S1. Geographic Regions Represented by Studies Included in Meta-Analysis 7

Figure S2. Risk of bias ratings in exposure measurement, participant/house selection and analysis domains. Solid blue represents a rating of "good", blue patch represents a rating of "adequate" while solid orange indicates a rating of "deficient" 8

Table S5. Sensitivity analysis exploring the influence of bias on the pooled median chemical concentrations 8

Table S6. Qualitative Summary of Extracted Data 9

Table S7. Quality assurance/ quality control measures 13

Table S8. Treatment of below-method of detection limit (MDL) values 14

Table S9. Measure of central tendency and measure of spread reported. 15

Table S10. Internal risk of bias assessment for studies included in meta-analysis. 16

Table S11: The weighted pooled median of *ortho*-phthalates and *ortho*-phthalate alternatives that were commonly detected (frequency ⩾ 50) in at least two studies. 19

Figure S3. Weighted pooled median phthalate and phthalate alternative concentrations in the indoor residential environment across sub-populations in the United States. The number depicted to the right of each bar graph represents the number of datasets that contributed to the calculation of the pooled median concentrations while the numbers depicted in brackets represent the total number of dust samples included in all datasets in the analysis for each phthalate or phthalate alternative. 20

Figure S4. Proportion of intake for children (3 to <6 years old) across three exposure pathways: ingestion, air inhalation, and dermal from air. The proportional contribution of the exposure pathways to the aggregate intake rate was similar for the 50th percentile for adults. On the x-axis, the abbreviation of each phthalate is in black while the abbreviation of each phthalate alternative is in blue. Within each grouping, the chemicals are arranged in order of increasing molecular mass 21

Equations used for modelling intake rate from dust 22

Table S12. Urine metabolites of detected phthalates and phthalate alternatives 24
Figure S5. Parent-metabolite mapping for phthalate and phthalate alternative metabolites of chemical substances identified in systematic review and measured in urine during the NHANES 2017-2018 cycle. Nodes that represent a parent chemical are displayed in dark blue and nodes that represent a metabolite measured in urine are displayed in teal. Metabolites not included in NHANES 2017-2018 year were not shown.. 24
Table S13. Excretion factors used to estimate daily intake from phthalate and phthalate alternative urinary metabolites 25
Table S14. Summary of data obtained from NHANES 2017-2018 Survey Year............................ 26
Figure S6. Correlation plot between molecular weight of phthalates and phthalates alternatives and their relative contribution to internal phthalate dose in the US population........................... 27

Table S1: List of ortho-phthalates and ortho-phthalate alternatives that are commonly detected (frequency ≥ 50) in the included studies

**Short chain (C1 – C3 backbone) and branched *ortho*-phthalates**						
**CASRN**	**Abbreviation**	**Substance name**	**Molecular Weight (g/mol)**	**Log Koa estimated**	**Log Kaw**	**Log Kow estimated**
131-11-3	DMP	Dimethyl phthalate	194.18	6.69	-5.51	*4.61*
84-69-5	DiBP	Diisobutyl phthalate	278.35	8.41	-5.91	4.46
84-66-2	DEP	Diethyl phthalate	222.24	7.02	-6.40	2.65
**Medium chain (C4 – C8 backbone) linear and branched *ortho*-phthalates incl. aromatics and cyclics**						
**CASRN**	**Abbreviation**	**Substance name**	**Molecular Weight (g/mol)**	**Log Koa estimated**	**Log Kaw**	**Log Kow estimated**
84-74-2	DBP	Dibutyl phthalate	278.35	8.63	-5.91	4.61
85-68-7	BBP	Benzyl butyl phthalate	312.36	9.02	-7.37	4.84
84-75-3	DHP	Dihexyl phthalate	334.45	9.80	-5.42	6.57
117-81-7	DEHP	Bis(2-ethylhexyl) phthalate	390.56	12.56	-4.93	8.39
117-84-0	DnOP	Dioctyl phthalate	390.56	12.08	-4.93	8.54
28553-12-0	DiNP	Diisononyl phthalate	418.61	13.59	-3.39	8.80
84-61-7	DCP	Dicyclohexyl phthalate	330.42	11.0	-5.90	5.78
**Long chain (C9-C18 backbone) linear and branched *ortho*-phthalates**						
**CASRN**	**Abbreviation**	**Substance name**	**Molecular Weight (g/mol)**	**Log Koa estimated**	**Log Kaw**	**Log Kow estimated**
84-76-4	DNP	Dinonyl phthalate	418.62	12.59	-5.27	9.40
84-77-5	DDP	Didecyl phthalate	446.67	11.87	-5.49	*9.05*
***Ortho*-Phthalate alternatives which include terephthalates & trimellitates**						
**CASRN**	**Abbreviation**	**Substance name**	**Molecular Weight (g/mol)**	**Log Koa estimated**	**Log Kaw**	**Log Kow estimated**
4654-26-6; 6422-86-2	DOTP, DEHT or DEHTP	Dioctyl terephthalate or bis(2-ethylhexyl terephthalate)	390.56	11.7	-4.98	8.43
77-90-7	ATBC	Acetyl tributyl citrate	402.48	12.10	-5.92	5.07
3319-31-1; 89-04-3	TOTM	Trioctyl trimellitate	546.79	16.24	-4.96	11.7
103-23-1	DEHA	Bis(2-ethylhexyl) adipate or di (2-ethylhexyl) adipate	370.58	12.87	-4.29	8.12
102-06-7	-	1,3-Diphenylguanidine	211.27	8.33	-6.81	2.70
88-19-7	-	2-Methylbenzenesulfonamide or o-toluenesulfonamide	171.21	6.75	-6.06	*0.84*

141-04-8	DIBA	Hexanedioic acid, bis(2-methylpropyl) ester or Diisobutyl adipate	258.36	7.85	-5.40	3.88
**Note**: The values used for the following parameters: Log Koa estimated, Log Kaw, and Log Kow estimated were obtained from EPA Epi Suite as seen in Mitro et al and Schossler et al. In the absence of data from EPA Epi Suite as reported in these studies, values from EPA CompTox Dashboard were used where experimental averages (italicized) were prioritized over predicted averages.						

Table S2: List of *ortho*-phthalates and *ortho*-phthalate alternatives that are commonly detected (frequency < 50) in the included studies.

**Medium chain (C4 – C8 backbone) linear and branched *ortho*-phthalates incl. aromatics and cyclics**						
**CASRN**	**Abbreviation**	**Substance name**	**Molecular Weight (g/mol)**	**Log Koa estimated**	**Log Kaw**	**Log Kow estimated**
131-18-0	-	Dipentyl phthalate	306.40	9.40	-5.44	*5.62*
131-16-8	-	Dipropyl phthalate	250.29	7.38	-5.34	*3.72*
3648-21-3	DHpP	Diheptyl phthalate	362.51	10.7	-4.99	7.49
**Phthalate alternatives which include terephthalates & trimellitates**						
120-55-8	-	Diethylene glycol dibenzoate or Di-o-benzoyldiethylene glycol	314.34	10.2	-6.32	3.30
**Note**: The values used for the following parameters: Log Koa estimated, Log Kaw, and Log Kow estimated were obtained from EPA Epi Suite as seen in Mitro et al and Schossler et al. In the absence of data from EPA Epi Suite as reported in these studies, values from EPA CompTox Dashboard were used where experimental averages (italicized) were prioritized over predicted averages.						

List of Abbreviations Used in Table S1 and S2

Abbreviation	Definition
CASRN	Chemical Abstracts Service Registry Number
Koa	Octanol-Air Partitioning Coefficients
Kaw	Air-Water Distribution Ratio
Kow	n-Octanol/Water Partition Coefficient

Formula for calculating Kaw:

$$\frac{Henry's\ Law\ constant}{R * T}$$

where:

R = 0.080206 atm/mol-K

T = 298 K (normal room temperature)

Table S3. Systematic Review Search Strategy (for the updated review)

Databases	Date of Search	Search strategy	Limits Placed on Search	Number of Records
Web of Science	May 6, 2022	phthalate* AND dust* AND human*	Date: Mar 1, 2015 - April 30, 2022 ; Language: English ; Countries/Regions: USA; Peer-Reviewed	93
PubMed	May 6, 2022	phthalate* AND dust*	Date: Mar 1, 2015 - April 30, 2022 ; Species: Human ; Language: English; Peer-Reviewed	132

Table S4. References of Studies Excluded At Full Study Stage in Systematic Review:

**Study**	**Reason for Exclusion**
Sears et al. 2020. Lowering Urinary Phthalate Metabolite Concentrations among Children by Reducing Contaminated Dust in Housing Units: A Randomized Controlled Trial and Observational Study.	Did not include dataset
Rudel et al. 2001. Identification of Selected Hormonally Active Agents and Animal Mammary Carcinogens in Commercial and Residential Air and Dust Samples.	Did not include information on timeline of collection

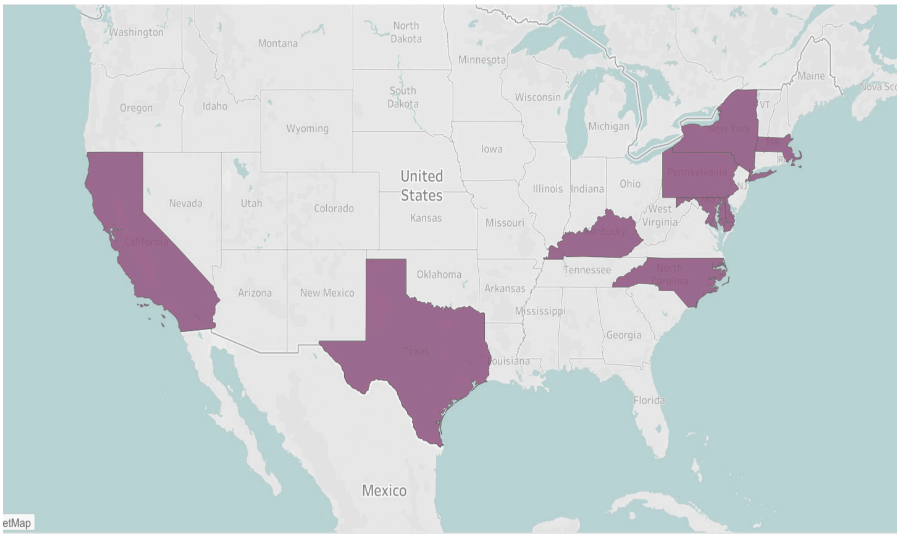

Figure S1. Geographic Regions Represented by Studies Included in Meta-Analysis.

Figure S2. Risk of bias ratings in exposure measurement, participant/house selection and analysis domains. Solid blue represents a rating of “good”, blue patch represents a rating of “adequate” while solid orange indicates a rating of "deficient”

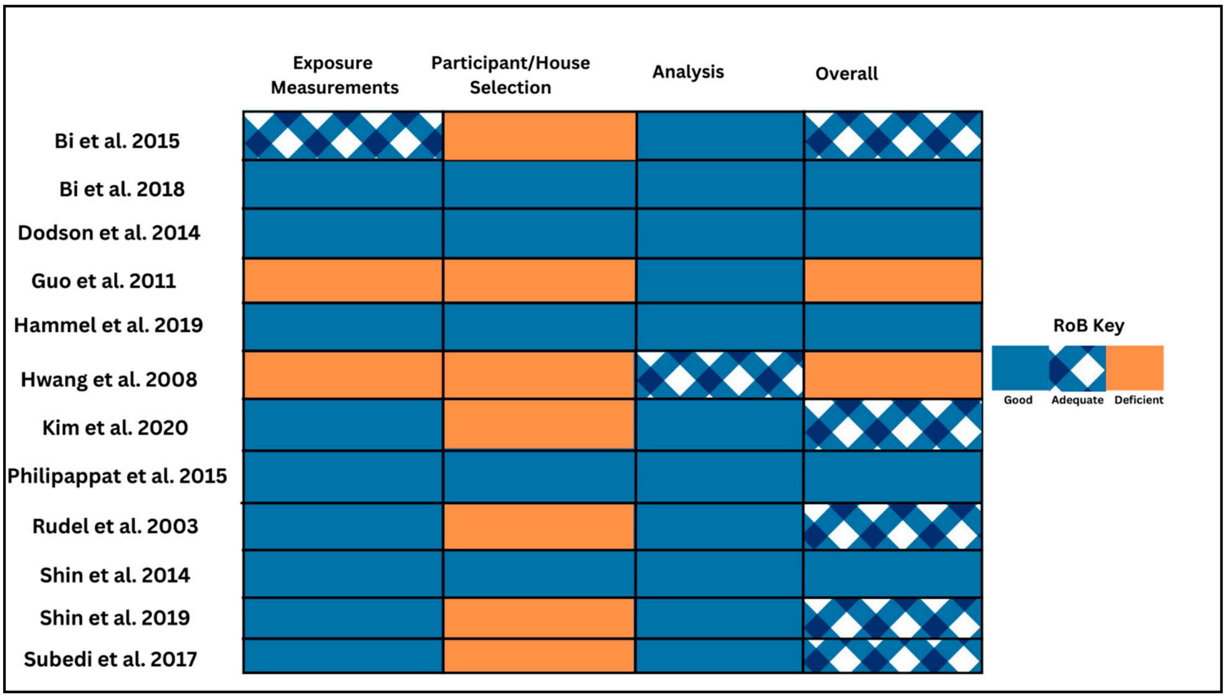

Table S5. Sensitivity analysis exploring the influence of bias on the pooled median chemical concentrations

Phthalates*	Pooled Medians of All Studies (ug/g)	Pooled Medians of Low Bias Studies (ug/g)	Relative Percent Change (%)
ATBC	271.00	271.00	0.00
BBP	13.64	13.64	0.00
DBP	10.30	10.30	0.00
DEHA	5.97	5.97	0.00
DEHP	140.00	140.00	0.00
DEHTP	133.65	133.65	0.00
DEP	1.94	1.94	0.00
DHP	1.10	1.10	0.00
DiBP	4.37	4.37	0.00
DiNP	78.75	78.75	0.00
DMP	0.07	0.07	0.00
DnOP	1.60	7.34	358.75

Table S6. Qualitative Summary of Extracted Data

Paper	*Ortho*-Phthalates & *ortho*-phthalate alternatives measured (frequency ≥50%)	Indoor Residential Environment	Study Length (Temporal Time Span)	Location of Study	Method	Sieve (μm)	Storage container	Storage temperature	Demographics Reported
**Bi et al. 2015**	DEHP, BBP, DBP, DiBP, DHP, DNP, DEP, DDP	Apartments (n=7), houses (n=10), student dormitories (n=5), house garage (n=3)	Mar 2013	Dover, Delaware	Study used vacuum	106	Ethyl acetate rinsed glass jars with steel caps	−21°C	No (X)
**Bi et al. 2018**	BBP, DEHP, DnOP	Settled dust in children's room including objects 30 cm above the floor.<br>---------------------<br>The total no. of dust samples =54	Jun 2014 – Sept 2014; Dec 2014 – Feb 2015	Central Texas (a part of the Healthy Homes investigation (HUD: TXHHU0023-13)	Study used vacuum		Cellulose thimbles in clean amber glass jars	−18°C	General description available
**Dodson et al. 2014**	BBP, DEHP,DBP,DHP, DnOP, DEP, DIBP, DEHA	Main living areas including windowsills, ceiling fans, upholstery, furniture.<br>---------------------<br>Dust samples =49; No repeat sampling	2006	Northern California – (Liberty and Atchison Village in Contra Costa County) in Richmond and Bolinas	Study used vacuum	150	Precleaned, certified glass jars with Teflon-lined lids	−4°C	Description of demographic composition

Paper	*Ortho*-Phthalates & *ortho*-phthalate alternatives measured	Indoor Residential Environment	Study Length (Temporal Time Span)	Location of Study	Method	Sieve (μm)	Storage container	Storage temperature	Demographics Reported
**Guo et al. 2011**	DMP, DEP, DiBP, DBP, DHP, BBP, DEHP, DnOP	Homes<br>-----------------<br>Dust samples (n=33)	Dec 2007 – Jan 2008	Albany, NY	Study used existing vacuum bags	2000	Clean aluminum foil	−4°C	No (X)
**Hammel et al. 2019**	DEP, DiBP, DBP, DMP, BBP, DEHP, DiNP, DEHTP or DOTP, TOTM, DEHA	Main living area or play area for child.<br>---------------------<br>Dust samples =188)	Sept 2014 – April 2016	Durham, NC	Study used vacuum	500	Cellulose thimble wrapped in aluminum foil	−20°C	Yes (✓)
**Hwang et al. 2008**	DEHP	Apartments<br>-------------------<br>No of dust samples (n = 10)	Fall 2014 (months not specified)	Davis, California	Study used existing vacuum bags	100	Glass jars pre-cleaned hexane	−20°C	No (X)
**Kim et al. 2020**	DEHP,BBP,DiBP, DBP,DnOP,DMP, DEHTP or DOTP, DEHA, ATBC	Main living area (except under furniture, between cushions and upholstered furniture)<br>------------------<br>Dust samples (n = 87)	Jul 2015 – May 2018 (22 months)	California<br>---------------<br>Northern California	Study used vacuum	150	Cellulose extraction thimble, wrapped in pre-cleaned aluminum foil	−20°C	Income of the underlying population was reported
**Philippat et al. 2015**	DMP, DBP, DEP, BBP, DEHP	Main living area carpets and rugs<br>---------------------<br>No. of dust samples = 145	2010 - 2011	California (Community not specified)	Study used vacuum	105	Glass jars pre-cleaned hexane	−20°C	Yes (✓)

Paper	*Ortho*-Phthalates & *ortho*-phthalate alternatives measured	Indoor Residential Environment	Study Length (Temporal Time Span)	Location of Study	Method	Sieve (µm)	Storage container	Storage temperature	Demographics Reported
**Rudel et al. 2003**	DEP, DBP, BBP, DEHP	Most frequently used rooms including hallways, windowsills, fans, and furniture	Jun 1999 – Sept 2001	Cape Cod, Mass	Study used vacuum	150	Certified glass jars with Teflon-lined lids	−4°C	No (X)
**Shin et al. 2014**	DEHP, DiNP, BBP, DBP,DiBP, DEP	Main living room but no samples were collected from under furniture or between cushions.<br>---------------------<br>(no. of dust samples = 30)	2009 - 2010	Northern California, SE Pennsylvania, NE Maryland (community location was not given)	Study used vacuum	150	Wrapped in pre-cleaned aluminum foil and placed in poly-propylene vials	−20°C	Yes (✓)*<br>---------------<br>Demographic information was provided for the larger survey pop. from which this data was drawn
**Shin et al. 2019**<br><br>**(targeted & non-target analysis)**	BBP, DEHP, DiBP, DBP, DEP, DMP, ATBC, DOTP, 1,3-diphenylguanidine toluene-2-sulfonamide	Main living room<br><br>Dust samples (n= 38)	May 2015 – August 2016	Sacramento and Fresno, CA	Study used vacuum	106	Polytetrafluoroethylene (PTFE) container	−20°C	No (X)

Paper	*Ortho*-Phthalates & *ortho*-phthalate alternatives measured	Indoor Residential Environment	Study Length (Temporal Time Span)	Location of Study	Method	Sieve (μm)	Storage container	Storage temperature	Demographics Reported
**Subedi et al. 2017**	DMP,DEP,DiBP, DBP,BBP,DEHP, DnOP, DIBA or hexanedioic acid, DEHA, ATBC	Homes<br>---------------------<br>(No. of dust samples = 11)	September 2016 – October 2016	•El Cerrito, CA<br>•San Diego, CA<br>•Waco, TX<br>•Murray, KY<br>•Silver Spring, MD<br>•Medway, MA	Study used vacuum	1400	Aluminum foil	−20°C	No (X)

*Phthalates noted are limited to the phthalates detected in the following indoor residential environments: apartment, house, house garage and student dormitory. No specific room where sampling was done was indicated in the paper.

Table S7. Quality assurance/ quality control measures

Paper	Analysis	Reference Material	Internal Standard	Lab blank	Field blank	Additional Accuracy/Precision methods
**Bi et al. 2015**	GC/MS equipped with AOC-i20 auto-sampler in SIM mode		✓	✓		Background was subtracted from all sample extracts, replicates (n=3) of samples
**Bi et al. 2018**	GC – FID system		✓	✓	✓	Matrix spiking, replicates
**Dodson et al. 2014**	GC/MS in selective ion-monitoring (SIM) mode		✓	✓		Analyzed solvent blanks (n=3), matrix spikes (n=3) and surrogate recoveries used to characterize accuracy, compound recovery from matrix, and extraction efficiency.
**Guo et al. 2011**	GS/MS in SIM mode		✓	✓		Matrix spikes, extraction efficiency tested by performing 4th extractions
**Hammel et al. 2019**	GC/MS in electron impact (EI) mode	✓	✓	✓		Laboratory blanks (n=6), house dust standard reference material (n=5)
**Hwang et al. 2008**	GC/MS in EI and SIM mode	✓	✓	✓		Replicates
**Kim et al. 2020**	GC/MS quadrupole time-of-flight (Q/TOF) in EI mode	✓	✓			Replicates
**Philippat et al. 2015**	GS/MS with glass capillary	✓	✓	✓		Matrix spiking, Replicates
**Rudel et al. 2003**	GS/MS in SIM mode		✓	✓		Matrix spiking, replicates (n = 4)
**Shin et al. 2014**	GC-EI/MS		✓	✓		Matrix spiking, replicates
**Shin et al. 2019**	LC-QTOF/MS and GC-QTOF/MS	✓	✓	✓		Replicates, calculation of absolute recovery
**Subedi et al. 2017**	GC/MS in SIM mode		✓	✓		Matrix spiking and matrix spike duplicate analysis, replicates (n=3), blank correction

Table S8. Treatment of below-method of detection limit (MDL) values

Paper	Treatment of MDL values				
	**No values fell below MDL**	**MDL/√2**	**MDL/2**	**Other treatment of MDL**	**Not reported**
**Bi et al. 2015**					✓
**Bi et al. 2018**			✓		
**Dodson et al. 2014**	✓				
**Guo et al. 2011**				0	
**Hammel et al. 2019**			✓		
**Hwang et al. 2008**	✓				
**Kim et al. 2020**		✓			
**Philippat et al. 2015**		✓			
**Rudel et al. 2003**				0	
**Shin et al. 2014**		✓			
**Shin et al. 2019**			✓		
**Subedi et al. 2017**				Determined to be minimum concentration of analytes	

Table S9. Measure of central tendency and measure of spread reported.

	Measure of central tendency reported			Measure of spread reported				
**Paper**	**Geometric Mean**	**Median**	**Unit reported**	**Variance**	**Standard Deviation**	**Range**	**IQR**	**Not reported**
**Bi et al. 2015**	✓	✓	mg/kg		✓	✓		
**Bi et al. 2018**	✓	✓	µg/g		✓	✓		
**Dodson et al. 2014**		✓	µg/g			✓		
**Guo et al. 2011**		✓	µg/g					
**Hammel et al. 2019**		✓	ng/g					
**Hwang et al. 2008**		To be calculated from raw values	µg/g			✓		
**Kim et al. 2020**	✓	✓	ng/g			✓	✓	
**Philippat et al. 2015**		✓	µg/g				✓	
**Rudel et al. 2003**	✓	✓	µg/g			✓		
**Shin et al. 2014**	✓	✓	µg/g		✓	Max; not min but can be estimated from LOD		
**Shin et al. 2019**		✓	ng/g				✓	
**Subedi et al. 2017**		To be calculated from raw value	µg/g			To be calculated	To be calculated	

Table S10. Internal risk of bias assessment for studies included in meta-analysis.

Study Name	Risk of Bias Domain	Rating	Authors' Reasoning
**Bi et al. 2015**	Exposure Measurement	Adequate	Methodology described, quality assurance and validation described. Measured dust level in an untraditional indoor environment space, i.e., the garage, and did not say where in the house the samples were collected from, or the surfaces sampled.
	Participant/Home Selection	Deficient	Little (only when + where) or no information on recruitment process beyond, selection strategy, sampling framework, and/or participation was included.
	Analysis	Good	Quantitative results presented and distribution for dust data was discussed.
	Conflict of Interest	Good	No conflict of interest disclosed or identified.
**Bi et al. 2018**	Exposure Measurement	Good	Methodology described in detailed, quality assurance and validation described. Minimal exposure media measurements were below the LOD.
	Participant/Home Selection	Good	Recruitment process was adequately described with minimal concern for selection bias based on description of recruitment process.
	Analysis	Good	Quantitative results were presented including details of how below-LOD measurements were treated. Distribution for dust data were discussed.
	Conflict of Interest	N/A	The presence or absence of conflict was not mentioned.
**Dodson et al. 2014**	Exposure Measurement	Good	Valid exposure measurement method. Very detailed and validated QA/QC were discussed.
	Participant/Home Selection	Good	Recruitment process was adequately described with minimal concern for selection bias based on description of recruitment process.
	Analysis	Good	Quantitative results were presented including details of how below-LOD measurements were treated. Distribution for dust data were discussed.
	Conflict of Interest	Good	No conflict of interest disclosed or identified.
**Study Name**	**Risk of Bias Domain**	**Rating**	**Reasoning**
**Guo et al. 2011**	Exposure Measurement	Deficient	Used an existing vacuum bag. Detailed and validated QA/QC were discussed.
	Participant/Home Selection	Deficient	Little or no information on recruitment process, selection strategy, sampling framework, and/or participation was included.
	Analysis	Good	Quantitative results were presented including details of how below-LOD measurements were treated. Distribution for dust data were discussed.
	Conflict of Interest	N/A	The presence or absence of conflict was not mentioned.

**Hammel et al. 2019**	Exposure Measurement	Good	Valid exposure measurement method. Detailed and validated QA/QC were discussed.
	Participant/Home Selection	Good	Recruitment process was adequately described with minimal concern for selection bias based on description of recruitment process.
	Analysis	Good	Quantitative results were presented including details of how below-LOD measurements were treated. Distribution for dust data were discussed.
	Conflict of Interest	Good	No conflict of interest disclosed or identified.
**Hwang et al. 2008**	Exposure Measurement	Deficient	Use of existing vacuum bag; make and model of vacuum cleaners/bags not identified. Validated analytical procedures.
	Participant/Home Selection	Deficient	Little or no information on recruitment process, selection strategy, sampling framework, and/or participation was included.
	Analysis	Adequate	Descriptive information provided but analysis of data was not robust.
	Conflict of Interest	N/A	The presence or absence of conflict was not mentioned.
**Kim et al. 2020**	Exposure Measurement	Good	See Shin 2019. Methodology described in detailed, quality assurance and validation described. Minimal exposure media measurements were below the LOD.
	Participant/Home Selection	Deficient	Little or no information on recruitment process, selection strategy, sampling framework, and/or participation was included.
	Analysis	Good	Quantitative results presented including details of how below-LOD measurements were treated. MDLs high but detection of chemicals were well above the MDLs.
	Conflict of Interest	Good	No conflict of interest disclosed or identified.
**Study Name**	**Risk of Bias Domain**	**Rating**	**Reasoning**
**Philippat et al. 2015**	Exposure Measurement	Good	Valid exposure measurement method. Detailed and validated QA/QC were discussed.
	Participant/Home Selection	Good	Recruitment process was adequately described with minimal concern for selection bias based on description of recruitment process.
	Analysis	Good	Quantitative results were presented but distribution for dust data were discussed briefly and showed table of variation.
	Conflict of Interest	Good	No conflict of interest disclosed or identified.
**Rudel et al. 2003**	Exposure Measurement	Good	Methodology described in detailed, quality assurance and validation described. Minimal exposure media measurements were below the LOD.
	Participant/Home Selection	Deficient	Little or no information on recruitment process, selection strategy, sampling framework, and/or participation was included.

	Analysis	Good	Methodology described in detailed, quality assurance and validation described. Minimal exposure media measurements were below the LOD.
	Conflict of Interest	N/A	The presence or absence of conflict was not mentioned.
**Shin et al. 2014**	Exposure Measurement	Good	Methodology described in detailed, quality assurance and validation described. Minimal exposure media measurements were below the LOD.
	Participant/Home Selection	Good	Recruitment process was adequately described with minimal concern for selection bias based on description of recruitment process but lacked response rate.
	Analysis	Good	Quantitative results presented including details of how below-LOD measurements were treated.
	Conflict of Interest	N/A	The presence or absence of conflict was not mentioned.
**Study Name**	**Risk of Bias Domain**	**Rating**	**Reasoning**
**Shin et al. 2019**	Exposure Measurement	Good	Methodology described in detailed, quality assurance and validation described. Minimal exposure media measurements were below the LOD.
	Participant/Home Selection	Deficient	Little or no information on recruitment process, selection strategy, sampling framework, and/or participation was included.
	Analysis	Good	Quantitative results presented including details of how below-LOD measurements were treated; missing data addressed.
	Conflict of Interest	Good	No conflict of interest disclosed or identified.
**Subedi et al. 2017**	Exposure Measurement	Good	Methodology described in detailed, quality assurance and validation described. Minimal exposure media measurements were below the LOD.
	Participant/Home Selection	Deficient	Little or no information on recruitment process, selection strategy, sampling framework, and/or participation was included.
	Analysis	Good	Quantitative results presented including details of how below-LOD measurements were treated.
	Conflict of Interest	N/A	The presence or absence of conflict was not mentioned.

Table S11: The weighted pooled median of *ortho*-phthalates and *ortho*-phthalate alternatives that were commonly detected (frequency ≥ 50) in at least two studies.

**Abbreviation**	**Classification**	**Pooled Estimate Weighted Median (ug/g)**	**Pooled Median Lower Bound of 95% Confidence Interval (ug/g)**	**Pooled Medians Upper Bound of 95% Confidence Interval (ug/g)**
ATBC	*Ortho*-phthalate alternative	271.00	7.97	7900.00
BBP	*Ortho*-phthalate	13.64	13.4	29.00
DBP	*Ortho*-phthalate	10.30	9.63	13.10
DEHA	*Ortho*-phthalate alternative	5.97	5.10	144.50
DEHP	*Ortho*-phthalate	140.00	118.57	242.00
DEHTP	*Ortho*-phthalate alternative	133.65	35.68	133.65
DEP	*Ortho*-phthalate	1.94	1.01	2.10
DHP	*Ortho*-phthalate	1.10	0.60	1.10
DiBP	*Ortho*-phthalate	4.37	1.91	4.40
DiNP	*Ortho*-phthalate	78.75	78.75	110.00
DMP	*Ortho*-phthalate	0.07	0.07	0.11
DnOP	*Ortho*-phthalate	1.60	0.28	27.10

Figure S3. Weighted pooled median phthalate and phthalate alternative concentrations in the indoor residential environment across sub-populations in the United States. The number depicted to the right of each bar graph represents the number of datasets that contributed to the calculation of the pooled median concentrations while the numbers depicted in brackets represent the total number of dust samples included in all datasets in the analysis for each phthalate or phthalate alternative.

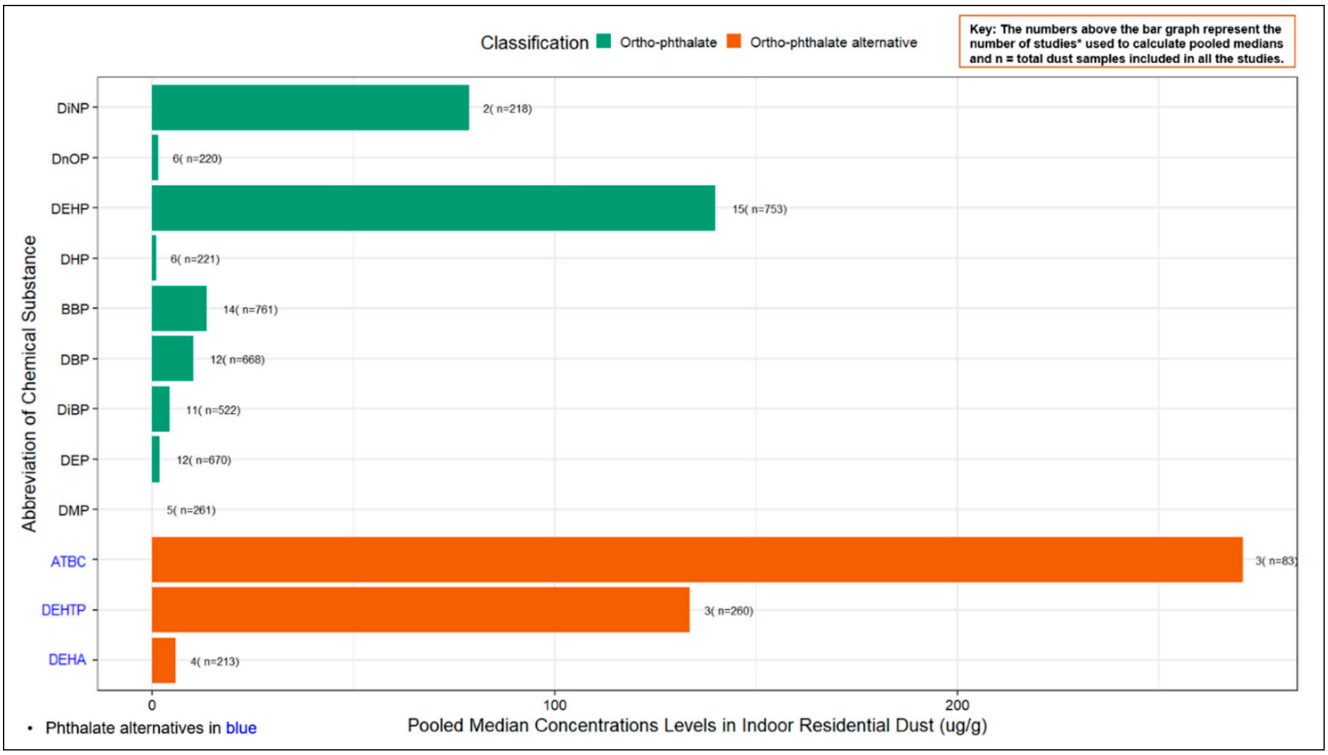

Figure S4. Proportion of intake for children (3 to <6 years old) across three exposure pathways: ingestion, air inhalation, and dermal from air. The proportional contribution of the exposure pathways to the aggregate intake rate was similar for the 50th percentile for adults. On the x-axis, the abbreviation of each phthalate is in black while the abbreviation of each phthalate alternative is in blue. Within each grouping, the chemicals are arranged in order of increasing molecular mass

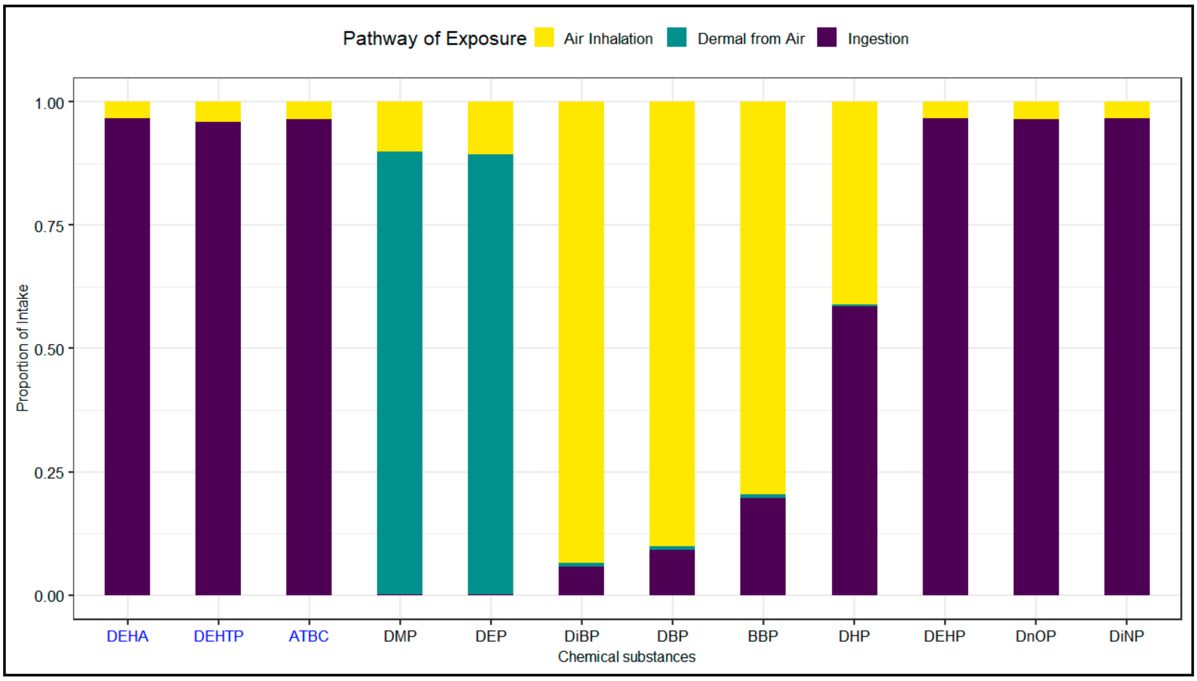

Equations used for modelling intake rate from dust

Assumed parameters are based on values seen in Mitro et al., Bekö et al., and Weschler et al.:

For DMP and DEP (ug/m³):

$$Concentration\ in\ gaseous\ phase = \left(\frac{Conc.in\ dust + 0.2381}{0.0092}\right) \div 1000$$

For all phthalates except DMP and DEP:

<u>Equation 1</u>

Dust to gaseous air concentration (ng/m³): $\frac{Dust\ concentration\left(\frac{ug}{g}\right)*rho_{dust}}{fom_{dust}*(10^{logKoa})}$

*where: Volume fraction of organic matter associated with settled dust (**fom_{dust}**) = 0.2*

*Density of dust (**rho_{dust}**) = 2 x 10^6 g/m³*

- To convert to ug/m³, the output was divided by 1000 to use in dermal from air intake formula.

<u>Equation 2</u>

Gaseous air concentration to total air concentration (ug/m³):

$$Gaseous\ air\ concencentration\ (\frac{ng}{g}) * \left(1 + \frac{TSP}{1 *\ 10^6} * \frac{fom_{part} * 10^{logKoa}}{rho_{part}}\right)$$

*where: Total suspended particles (**TSM**)/ug/m³ = 20*

*Volume fraction of organic matter associated with airborne particles (**fom_{part}**) = 0.4*

*Density of airborne particles (**rho_{part}**)/g/m³ = 1 x 10^6*

<u>Equation 3</u>

Indoor air transdermal permeability: $\frac{1}{\frac{1}{vd}+\frac{1}{kp_b}}$

*<u>where</u>: Mass-transfer coefficient between bulk air and skin surface (**vd**)/cm/hr = 600*

***kp_b** is a permeability coefficient that describes the transport of a gas-phase SVOC from the boundary layer at the skin surface (b) through the stratum corneum/viable epidermis composite to dermal capillaries (m/h) = $\boldsymbol{kp_w} * 10^{abs(logKaw)}$*

and

***kp**w is a permeability coefficient through the stratum corneum/viable epidermis composite of SVOC when the species concentration is measured in water in contact with skin (m/h) = $\frac{\boldsymbol{kp_{cw}}}{\boldsymbol{1+B}}$*

and

kp *$_{cw}$ is a permeability coefficient through stratum corneum (c) of an SVOC when the species concentration is measured in water (w) in contact with skin (m/h) =*

$$\left(10^{0.7*logKow-0.0722*molecular\ weight^{\frac{2}{3}}-5.252}\right)*3600$$

and

*Ratio of stratum corneum permeability to viable epidermis permeability **(B)** =*

$$kp_{cw} * \frac{molecular\ weight^{0.5}}{2.6}$$

References:

1. Bekö, G. et al. Children's Phthalate Intakes and Resultant Cumulative Exposures Estimated from Urine Compared with Estimates from Dust Ingestion, Inhalation and Dermal Absorption in Their Homes and Daycare Centers. PLOS ONE 8, e62442 (2013).
2. Mitro, S. D. et al. Consumer Product Chemicals in Indoor Dust: A Quantitative Meta-analysis of U.S. Studies. *Environ Sci Technol* **50**, 10661–10672 (2016).
3. Weschler, C. J. & Nazaroff, W. W. SVOC exposure indoors: fresh look at dermal pathways. Indoor Air 22, 356–377 (2012).

Table S12. Urine metabolites of detected phthalates and phthalate alternatives

Parent Compound	Metabolite(s)
Diethyl phthalate (DEP)	Mono-ethyl phthalate (MEP)
Diisobutyl phthalate (DiBP)	Mono-isobutyl phthalate (MiBP)<br>Mono-2-methyl-2-hydroxypropyl phthalate (MHiBP)
Dibutyl phthalate (DBP)	Mono (3-carboxylpropyl) phthalate (MCPP)<br>Mono-n-butyl phthalate (MBP)<br>Mono-3-hydroxybutyl phthalate (MHBP)
Dioctyl phthalate (DnOP)	Mono (3-carboxylpropyl) phthalate (MCPP)
Benzylbutyl phthalate (BBP)	Mono-benzyl phthalate (MBP)
Bis (2-ethylhexyl) phthalate (DEHP)	Mono(2-ethylhexyl) phthalate (MEHP)<br>Mono(2-ethyl-5-carboxypentyl) phthalate (MECPP)<br>Mono(2-ethyl-5-hydroxyhexyl) phthalate (MEHHP)<br>Mono(2-ethyl-5-oxohexyl) phthalate (MEOHP)
**Parent Compound**	**Metabolite(s)**
Bis (2-ethylhexyl) terephthalate (DEHTP/DOTP)	Mono(2-ethyl-5-hydroxyhexyl) terephthalate (MEHHTP)<br>Mono(2-ethyl-5-carboxypentyl) terephthalate (MECPTP)

Figure S5. Parent-metabolite mapping for phthalate and phthalate alternative metabolites of chemical substances identified in systematic review and measured in urine during the NHANES 2017-2018 cycle. Nodes that represent a parent chemical are displayed in dark blue and nodes that represent a metabolite measured in urine are displayed in teal. Metabolites not included in NHANES 2017-2018 year were not shown

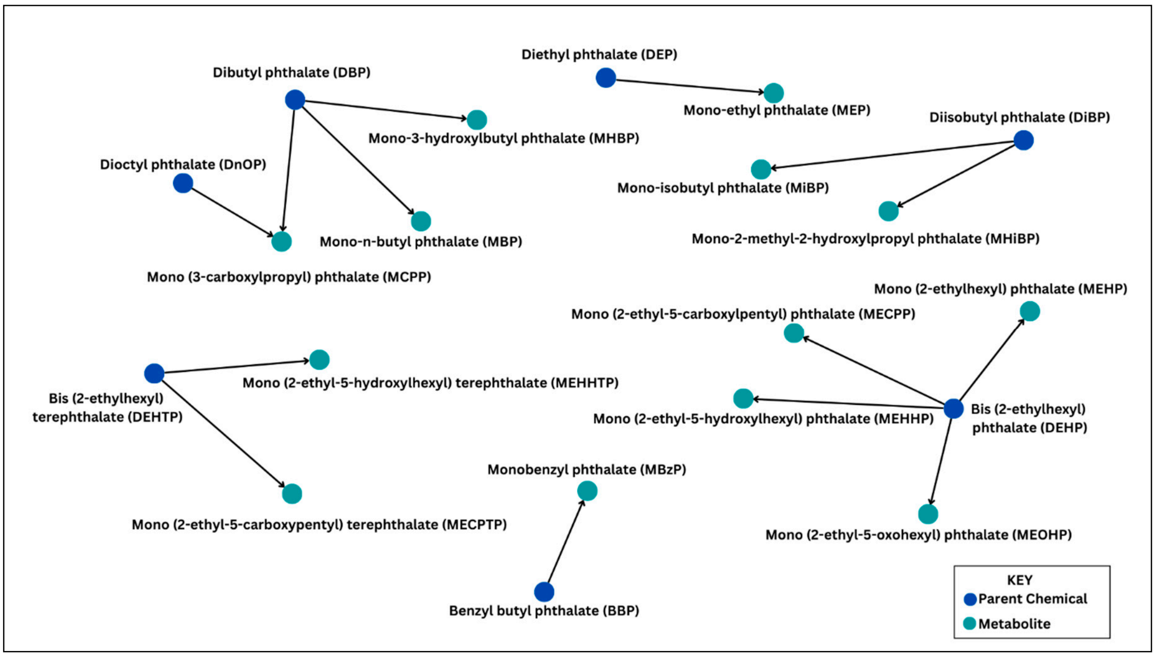

Table S13. Excretion factors used to estimate daily intake from phthalate and phthalate alternative urinary metabolites

Phthalate/Phthalate Metabolite	Molecular Weights	Excretion factor (Fue)	References
MBP	222	0.690	Anderson et al. (2001)
MHBP	238	0.069	Lee et al. (2021); Koch et al. (2011)
MiBP	222	0.703	Koch et al. (2011); Anderson et al. (2001)
MHiBP	238	0.195	Lee et al. (2021); Koch et al (2011)
MEP	194	0.69	Wang et al. (2014)
MBzP	256	0.73	Anderson et al. (2001)
MEHP	278	0.062	Anderson et al. (2001)
MEOHP	292	0.109	Anderson et al. (2001)
MECPP	308	0.132	Anderson et al. (2001)
MEHHP	294	0.149	Anderson et al. (2001)
MEHHTP	294	0.018	Lessmann et al. (2016)
MECPTP	308	0.13	Lessman et al. (2016)
MCPP	308	0.139	Lee et al. (2021); Anderson et al (2011)

Table S14. Summary of data obtained from NHANES 2017-2018 Survey Year

Metabolite Abbreviation	Age category	Median Creatinine adjusted phthalate concentration (ug/g)	Creatinine excretion rate (g/day)	Median Urinary excretion rate (ug-kg-day)	Median Weight (kg)
MBP	Adult	7.552	1.535	0.144	80.6
	Child	19.492	0.284	0.315	17.6
MCPP	Adult	0.878	1.535		80.6
	Child	2.658	0.284		17.6
MHBP	Adult	0.6155	1.535	0.012	80.6
	Child	2.304	0.284	0.037	17.6
MEP	Adult	22.744	1.535	0.433	80.6
	Child	26.757	0.284	0.432	17.6
MiBP	Adult	5.884	1.535	0.112	80.6
	Child	16.479	0.284	0.266	17.6
MHiBP	Adult	1.844	1.535	0.035	80.6
	Child	6.556	0.284	0.106	17.6
MEHP	Adult	0.501	1.535	0.010	80.6
	Child	1.595	0.284	0.026	17.6
MECPP	Adult	5.971	1.535	0.114	80.6
	Child	22.858	0.284	0.369	17.6
MEHHP	Adult	3.776	0.072	0.100	80.6
	Child	12.227	0.197	0.203	17.6
MEOHP	Adult	2.371	1.535	0.045	80.6
	Child	8.683	0.284	0.140	17.6
MBzP	Adult	2.459	1.535	0.047	80.6
	Child	8.860	0.284	0.143	17.6
MEHHTP	Adult	5.269	1.535	0.100	80.6
	Child	12.581	0.284	0.203	17.6
MECPTP	Adult	17.212	1.535	0.328	80.6
	Child	80.270	0.284	1.296	17.6

Figure S6. Correlation plot between molecular weight of phthalates and phthalates alternatives and their relative contribution to internal phthalate dose in the US population

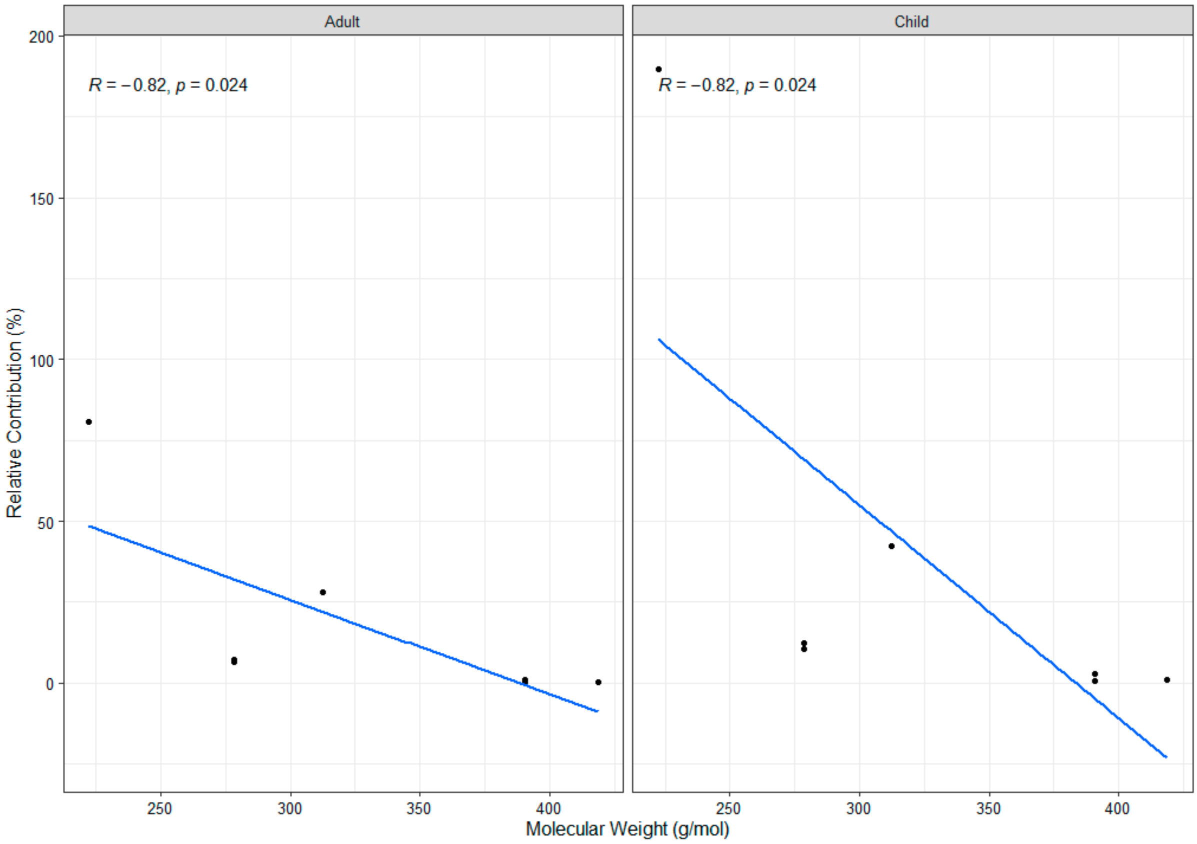

Supplement: Supplementary file 1 [file ijerph-20-06589-s001.zip › ijerph-2479015-supplementary.pdf]
